# Nanosystems for delivery of indolicidin peptide

**DOI:** 10.3389/fmedt.2026.1845120

**Published:** 2026-07-20

**Authors:** José Gregorio Martín Bedoya, Katia Conceição

**Affiliations:** Laboratory of Peptide Biochemistry, Federal University of São Paulo, UNIFESP, São José dos Campos, Brazil.

**Keywords:** antimicrobial peptides (AMPs), indolicidin, multidrug-resistant bacteria, nanoparticle delivery systems, pharmacokinetics

## Abstract

The emergence of multidrug-resistant pathogens has necessitated the search for novel therapeutic alternatives such as antimicrobial peptides (AMPs). Indolicidin (Ind) represents a highly promising candidate owing to its broad-spectrum activity and multimodal mechanisms; however, its clinical translation is limited by rapid proteolytic degradation and intrinsic host cytotoxicity. To overcome these pharmacological bottlenecks, this review places nanocarrier based delivery of Ind at its analytical center, rigorously evaluating the integration of Ind into nanoscale delivery systems as a transformative therapeutic strategy. We critically analyze how diverse organic and inorganic platforms fundamentally reconfigure Ind's mechanism of action, including the bidirectional relationship between nanoparticle-generated ROS and peptide-mediated membrane disruption. Furthermore, we discuss emerging bio-inspired approaches utilizing stimuli-responsive hydrogels, lipid cubic phases, and liposomes for programmed intracellular release. A critical assessment of long-term genotoxicological and ecotoxicological safety profiles is subsequently provided to address mandatory regulatory prerequisites. This integrated analysis establishes a comprehensive mechanistic and translational framework for the rational development of next-generation Ind-based antimicrobial nanomedicines, balancing structural tethering with supramolecular assembly to effectively combat antibiotic-resistant infections.

## 1 Introduction

The alarming proliferation of multidrug-resistant (MDR) pathogens has severely compromised the efficacy of conventional antibiotic therapies, positioning antimicrobial resistance (AMR) as one of the most critical threats to global public health. According to the World Health Organization (WHO), AMR is responsible for approximately 1.27 million direct deaths per year, a figure projected to escalate to 10 million annually by 2050 unless appropriate interventions are implemented (196). Consequently, antimicrobial peptides (AMPs) have emerged as a compelling class of next-generation antimicrobial agents (1, 2, 186, 197). Among these, indolicidin (Ind), a unique 13-residue cationic peptide isolated from bovine neutrophils, occupies a prominent position due to its exceptionally high tryptophan content (39%) and its sophisticated dual mechanism of action. Unlike many AMPs that rely solely on membrane lysis, Ind is capable of translocating across the lipid bilayer to inhibit DNA topoisomerase I and to disrupt protein synthesis, thereby minimizing the likelihood of resistance development (3, 4).

The most advanced Ind analog, omiganan (MBI-226), a 12-residue synthetic derivative, progressed through Phase III clinical trials for catheter-related bloodstream infection prevention and Phase II trials for rosacea without achieving regulatory approval, impeded by rapid plasma degradation and dose-limiting toxicity at systemically relevant concentrations (5, 6). Yet the clinical potential of Ind has remained systematically unrealized: this clinical pathway underscores a compelling translational necessity, as the inherent pharmacological attributes of Ind are inadequate for clinical efficacy in the absence of a delivery mechanism proficient in safeguarding the peptide from proteolytic degradation, thereby constricting its therapeutic range and directing it to the infection site. Nanocarrier technology represents the most mechanistically sophisticated and experimentally validated approach available to meet this imperative (168, 180, 190).

Although Ind exhibits potent broad-spectrum activity, with minimum inhibitory concentrations (MICs) as low as 1.56 *μ*g.mL^−1^ against susceptible Gram-negative pathogens, its therapeutic window is critically narrow owing to concentration-dependent hemolysis and cytotoxicity that coincide with therapeutically relevant concentrations. To circumvent these barriers and bypass rapid proteolytic degradation, nanotechnology has emerged not merely as a sequestration method, but as a fundamentally transformative engineering strategy (166, 168, 169). Recent evidence demonstrates that peptide-conjugated nanoparticle platforms not only optimize targeted delivery but also open new avenues for multifunctional applications, including biosensing and real-time imaging of infection sites (7).

Nanoscale delivery systems provide versatile scaffolds that stabilize Ind and enable precise control over its biological activity. Rather than producing a merely additive effect, incorporation into nanocarriers can fundamentally reshape the peptide's pharmacodynamics (193). Depending on the design of the system, nanotechnology may either restrict Ind's conformational freedom through surface conjugation or promote its controlled release within the infectious microenvironment. In both cases, the result is a shift in activity away from canonical intracellular targets and toward more localized membrane disruption.

The review strategy was based on systematic literature searches conducted in PubMed, Scopus, and Web of Science from 2010 to 2026. Search terms included “indolicidin AND nanoparticle,” “indolicidin AND liposome,” “indolicidin AND gold,” “indolicidin AND hydrogel,” and “indolicidin AND gene delivery.” Eligible studies were original research articles reporting experimental data on indolicidin or its analogs, including omiganan, incorporated into or conjugated with nanocarrier systems, as well as studies describing antimicrobial, antifungal, antiviral, or gene-delivery outcomes. We excluded studies that evaluated Ind only in its free form, purely computational studies without experimental validation, and conference abstracts lacking full datasets. Reference lists of the selected articles were also screened manually to identify additional relevant studies. Overall, our aim was to assess how different nanoplatforms influence therapeutic efficacy, synergistic interactions, and the safety profiles required for next-generation antimicrobial applications (166). While Ind serves as the central case study, many principles discussed herein apply broadly to cationic AMP-nanocarrier systems (190).

## 2 Physicochemical properties and dual mechanism of action

**Figure 1:**
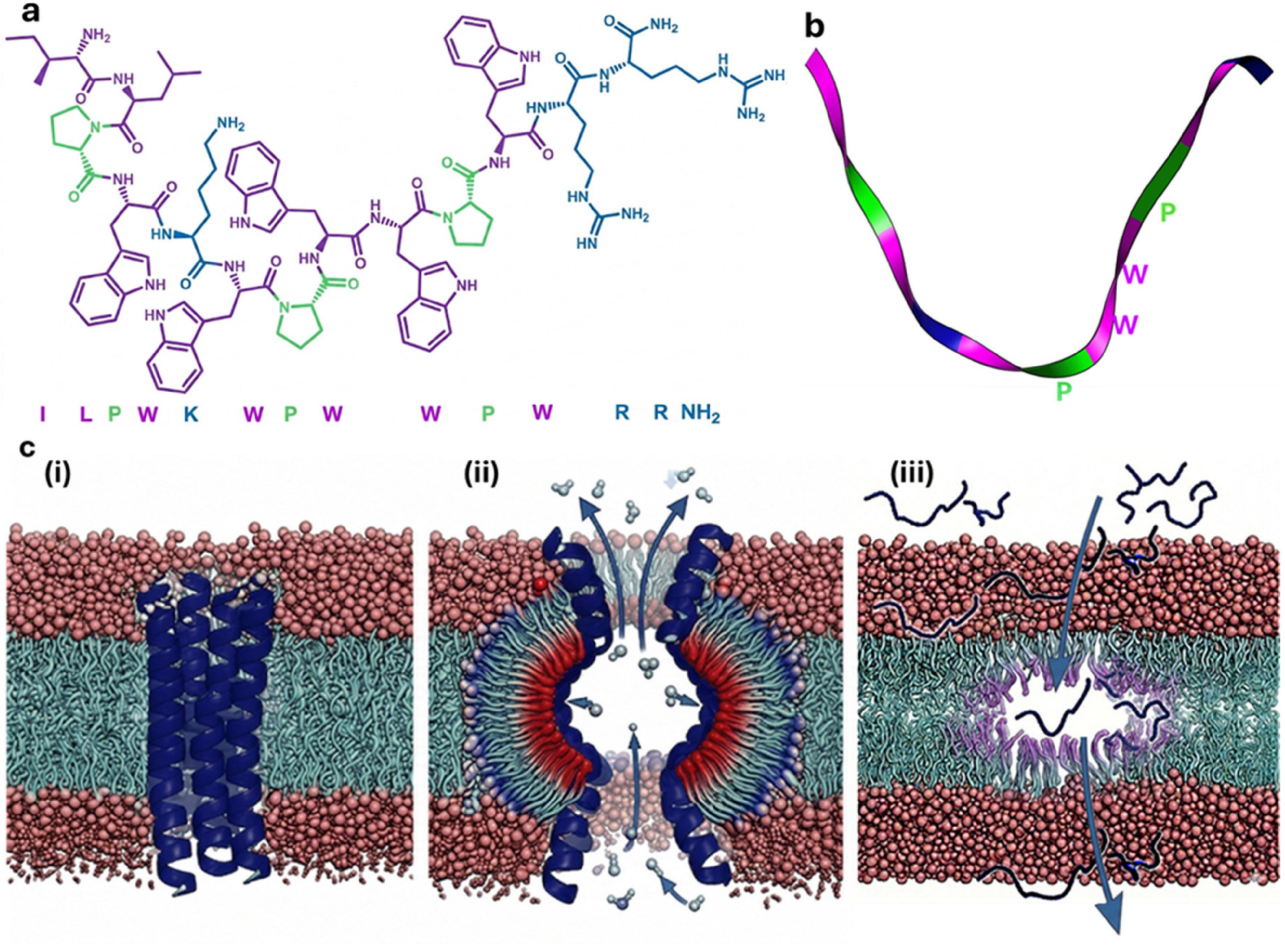
Structural features of indolicidin and proposed mechanisms of membrane interaction. **(a)** Chemical structure and amino acid sequence of indolicidin (Ind), with residues color-coded according to their physicochemical properties. **(b)** Three-dimensional ribbon representation of Ind (PDB ID: 1G89), highlighting the spatial distribution of aromatic hydrophobic (magenta), aliphatic hydrophobic (green), and positively charged (blue) residues that contribute to its amphipathic organization. **(c)** Representative models of antimicrobial peptide interactions with biological membranes, including the barrel-stave (i), toroidal pore (ii), and membrane permeation/translocation (iii) mechanisms.

Isolated from bovine neutrophils, indolicidin (Ind) is a remarkably short 13-residue peptide (ILPWKWPWWPWRR-NH_2_) that represents one of the most structurally compact yet functionally multivalent members of the cathelicidin family (Figure 1A). Ind is distinguished by a high tryptophan content and a net charge of +4, properties that confer strong membrane affinity and drive ionic interactions with anionic nanoparticle surfaces, making Ind an inherently “ adhesive” molecule for which loading efficiency and controlled release from nanocarriers must be carefully engineered. This high aromatic content, combined with the presence of proline residues at positions 3 and 7, prevents adoption of a classical *α*-helical or *β*-sheet secondary structure, resulting in an elongated, wedge-like topology upon interaction with hydrophobic interfaces (Figure 1B) (8, 9). Critically for nanocarrier design, the absence of a stable secondary structure in solution means that Ind's amphipathic conformation is induced only upon interaction with lipid or hydrophobic interfaces, a property that renders its encapsulation efficiency highly sensitive to the physicochemical nature of the carrier matrix.

While Ind remains disordered in aqueous solutions, it transitions into a structurally defined, amphipathic wedge conformation upon partitioning into lipid bilayers (10, 11). In this state, the tryptophan residues bury deeply into the hydrophobic core, while the cationic lysine and arginine residues remain anchored at the membrane–water interface (12–14, 179). Molecular dynamics (MD) simulations indicate that this specific orientation facilitates preferential partitioning into anionic bacterial membranes over zwitterionic mammalian membranes, providing a structural basis for Ind's selectivity profile (15, 16, 192).

Unlike pore-forming AMPs such as magainin (toroidal pore model) or alamethicin (barrel-stave model), which create stable transmembrane channels, Ind-induced membrane permeabilization is characterized by transient, stochastic lipidic defects that result in gradual, incomplete leakage of fluorescent reporters (e.g., calcein) rather than complete lysis (Figure 1C**)** (10, 17). Differential scanning calorimetry (DSC) measurements further indicate significant disruption of the lipid phase transition without complete solubilization of the bilayer (3, 17, 18). Subsequent full translocation across the bilayer encounters a relatively low free-energy barrier, explaining Ind's ability to cross membranes without requiring dedicated transporters (18). This highly specific transient permeabilization allows the peptide to effectively translocate across both bacterial and fungal membranes without causing the massive, immediate lysis typical of purely membranolytic peptides (19–21).

Once translocation is complete, Ind targets the intracellular machinery. Sublethal exposure induces profound bacterial filamentation, a hallmark of DNA synthesis inhibition, while leaving the bacterial envelope relatively intact (198). Specifically, the central PWWP motif of the internalized peptide intercalates into B-type DNA duplexes, physically blocking topoisomerase I activity through steric occlusion of its DNA-binding site (4, 22, 163). This leads to impaired relaxation of torsional stress, replication fork stalling, and ultimately bacterial cell death. Furthermore, Ind has been shown to covalently crosslink abasic sites in single- and double-stranded DNA, and to inhibit HIV-1 integrase, collectively establishing Ind's intracellular DNA targeting as a multi-modal mechanism (4). Crucially, because topoisomerase I is mechanistically and structurally distinct from topoisomerase II, the canonical target of fluoroquinolones, Ind retains full activity against fluoroquinolone-resistant clinical isolates (4, 23). This retention of activity against fluoroquinolone-resistant strains is a particularly compelling argument for Ind nanocarrier development: unlike combination therapies that merely circumvent resistance, an Ind-nanocarrier system designed to deliver active free peptide intracellularly can attack a target that existing resistance mechanisms do not protect. This duality of transient membrane penetration coupled with conformation-dependent intracellular inhibition renders Ind an extraordinarily difficult target for classical structural bacterial resistance mechanisms (20, 186).

Beyond its direct microbicidal mechanisms, Ind functions as a potent immunomodulatory agent. It stimulates monocyte and macrophage activation, enhancing cytokine production (IL-6, IL-12, TNF-α) and promoting neutrophil recruitment (24, 199). Furthermore, Ind interferes with quorum sensing signaling pathways and degrades components of the extracellular polymeric substance (EPS) of biofilms, which are notoriously resistant to conventional antibiotics (25, 26). However, the clinical translation of native Ind is hindered by its high proteolytic susceptibility. To address this, synthetic analogs like LD-indolicidin (incorporating D-amino acids) have been developed to enhance enzymatic stability while maintaining immunoregulatory efficacy (12). In murine models of intranasal influenza vaccination, nanoformulations of Ind and LD-indolicidin achieved an antigen dose-sparing effect of 10- to 40-fold, positioning the peptide as a high-efficiency mucosal adjuvant (12, 24, 183).

Despite this multifaceted profile, Ind is counterbalanced by significant pharmacological constraints (Table 1). While it exhibits potent activity (MICs as low 1.56 *μ*g.mL^−1^ for *Bacillus subtilis* and *Escherichia coli*), its therapeutic index remains narrow. Cytotoxic thresholds for human cell lines (e.g., PBMCs IC_50_ of 50.3 μg.mL^−1^) often overlap with the concentrations required to inhibit more resistant pathogens like *Salmonella enterica* or *Candida albicans* (MICs up to 64–100 μg.mL^−1^). This narrow therapeutic index is not just an academic concern; it is the primary pharmacological reason why nanocarrier encapsulation is necessary rather than merely advantageous for Ind, a distinction that has not been adequately foregrounded in prior literature. Consequently, free Ind exhibits moderate hemolytic activity and concentration-dependent cytotoxicity in mammals. This profile, compounded by its high susceptibility to proteolysis, contributes to an unfavorable therapeutic margin that severely limits its systemic clinical application. These limitations, systematically quantified in Table 1, collectively position Ind as an ideal molecular scaffold for optimization through nanoformulation strategies as the primary translational intervention, with sequence engineering playing a complementary role (52).

**Table 1:** Minimum inhibitory concentration (MIC), cytotoxicity and hemolytic activity of indolicidin against clinically relevant microorganisms.

Microorganism	Indolicidin MIC (µg.mL^−1^)	References
*Staphylococcus aureus*	2-64	(27–29)
*Methicillin resistant S. aureus*	8–31.25	(30, 31)
*Staphylococcus epidermidis*	4–20	(32–34)
*Streptococcus pneumoniae*	15.62–31.25	(31)
*Bacillus subtilis*	1.56 −20	(27, 33–35)
*Bacillus cereus*	10	(29)
*Escherichia coli*	1.56–31.25	(27, 31, 34, 35)
*Pseudomonas aeruginosa*	5–31.25	(31, 36, 37)
*Klebsiella pneumoniae*	2–19	(36, 198)
*Acinetobacter baumannii*	8–64	(38)
*Salmonella typhimurium*	4–64	(32–34, 39)
*Salmonella enterica*	7.63–100	(29, 36)
*Candida albicans*	32–64	(40–42)
*Candida krusei*	10–25	(43, 44)
*Candida glabrata*	60–90	(43, 44)
*Candida tropicalis*	16–150	(43–45)
*Cryptococcus neoformans*	4–45	(43, 44)
*Pneumocystis jirovecii*	IC_50_, 38	(46)
Antiviral		
Herpes simplex virus (HSV)	EC_50,_ 9.5–55	(47–49)
Parasite (%Viable)		
*Cryptosporidium parvum*	95.3 (37)	(44)
*Trypanosoma brucei*	BSF, 125–250 PCF, 250–500	(50)
*Leishmania donovani*	5 × 10^−5^	(207)
*Giardia lamblia* Trophozoitos Cysts	5 (40); 50 (99) 50 (34–66)	(208)
Cytotoxicity (%Viable)		
HaCaT	200 (11.3)	(37)
WRL-68	62.5	(31)
NL-20	62.5	(31)
Human dermal fibroblasts (HDF)	95.3 (100)	(35)
PBMC	IC_50_, 50.3	(51)
NIH3T3 fibroblast	IC_50,_ 24,35	(41)
Anticancer (%Viable)		
Human neuroblastoma SH-SY5Y	IC_75,_ 13.1	(52)
RAW 264.7	122 (80)	(53)
HEp-2	122 (85)	(53)
Jurkat	IC_50,_ 26.1	(51)
ME-180	100 (60.5)	(49)
J774	LD_50_, 20.96	(36)
Hemolytic activity (>5%)		
Sheep red blood	244	(53)
Human erythrocytes	32–47.7 MIH_50,_ 112.5–200	(31, 54) (27)
Rat erythrocytes	47.6	(36)
*Galleria mellonella*	LD_50_, 61	(53)

## 3 Mechanistic implications of the “bound” vs. “free” states in nanoconjugation

The integration of Ind into nanocarrier systems necessitates a rigorous thermodynamic evaluation of whether the peptide exerts its antimicrobial effect as a liberated entity or as a surface-anchored complex. This distinction is not merely semantic; it constitutes a fundamental pharmacodynamic variable that determines which molecular targets the peptide can access and, therefore, dictates the spectrum and magnitude of its bactericidal activity. The functional performance of these systems is governed by three distinct physicochemical scenarios: (i) physical encapsulation with post-release free-peptide activity, (ii) non-cleavable covalent surface conjugation, and (iii) conjugation via stimuli-responsive cleavable linkers. Each scenario fundamentally reconfigures the Ind mechanism, as detailed in Sections 3.1–3.3.

**Figure 2:**
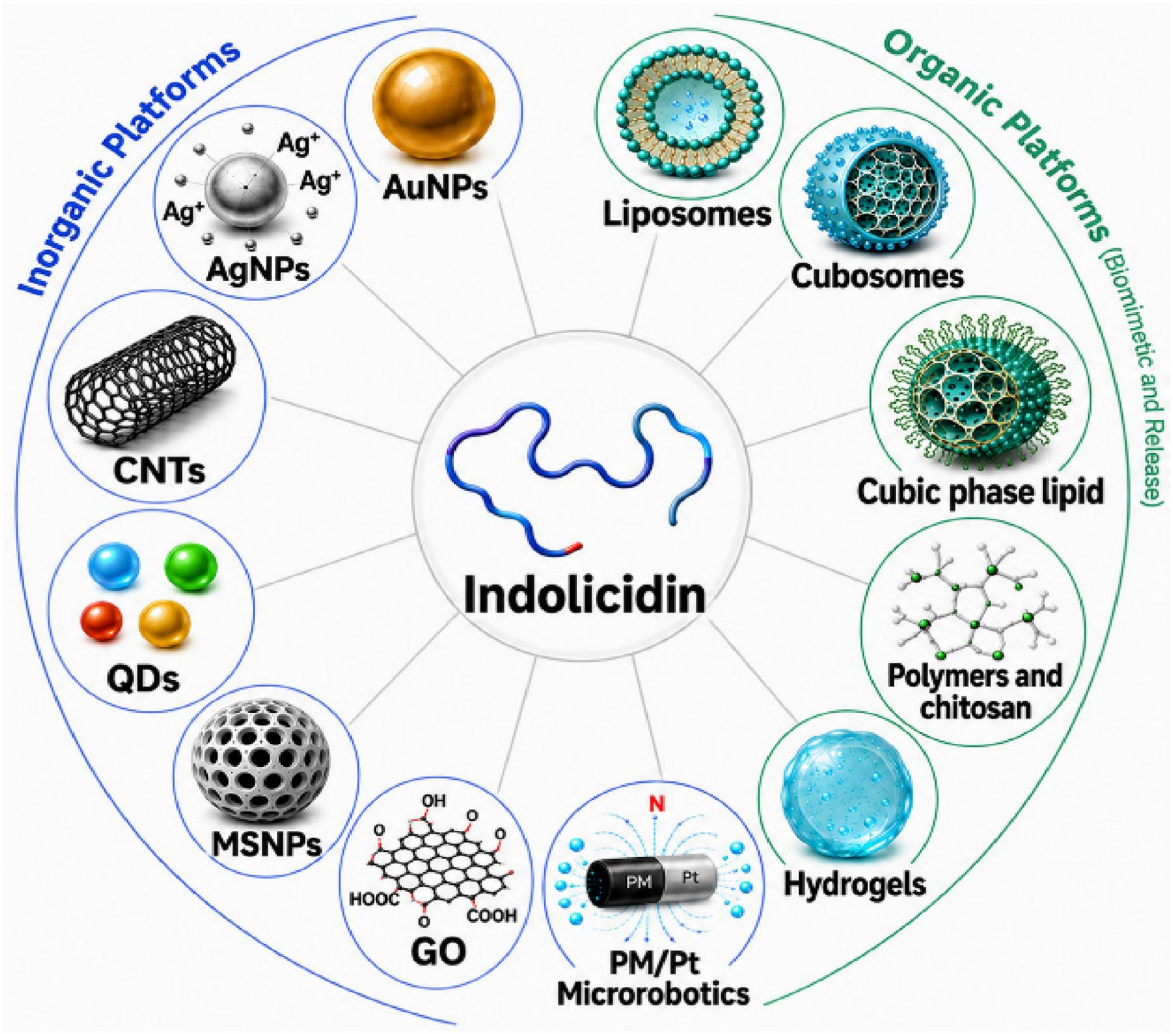
Nanomaterials used for optimizing the delivery of the peptide AMP indolicidin, AuNP (gold nanoparticle), AgNP (silver nanoparticle), CNTs (carbon nanotubes), QDs (quantum dots), MSNPs (mesoporous silica nanoparticles), GO (graphene oxide). (created with AI).

To navigate these scenarios, it is essential to classify the diverse array of available platforms into inorganic and organic nanocarriers, further distinguishing them by their static or active functionality (Figure 2). The versatility of Ind stems from its unique amphipathic structure and tryptophan-rich sequence, allowing it to interact dynamically with a wide array of delivery systems (166, 172). Within the inorganic category, carbon-based nanomaterials such as graphene oxide (GO) have emerged as a disruptive platform; their two-dimensional architecture facilitates high-density peptide loading via strong π- π stacking and electrostatic interactions, while simultaneously inducing physical membrane stress. Furthermore, the development of paramagnetic/platinum (PM/Pt) microrobots marks a crucial transition from passive diffusion to active transport. Unlike static metallic nanoparticles (e.g., AuNPs, AgNPs), these autonomous systems utilize catalytic or magnetic propulsion to mechanically penetrate the extracellular polymeric substance (EPS), delivering Ind directly to bacterial clusters. Conversely, organic platforms such as liposomes, cubosomes, and hydrogels exploit Ind's amphiphilicity to provide biomimetic encapsulation, shielding the peptide from proteolytic enzymes while enabling a biocompatible interface for controlled release

### 3.1 Encapsulation in degradable nanomaterials

In systems where Ind is physically encapsulated, the nanomaterial serves as a protective vehicle that facilitates initial translocation across biological barriers (181). The encapsulation process is governed by electrostatic and hydrophobic interactions between Ind's cationic residues and the carrier matrix. In the case of liposomal formulations, the cationic character of Ind (+4 net charge) drives its preferential association with negatively charged lipid headgroups at the inner leaflet, whereas its tryptophan-rich hydrophobic core partitions into the bilayer interior, effectively anchoring the peptide in a transmembrane orientation during transport (55, 164).

**Figure 3:**
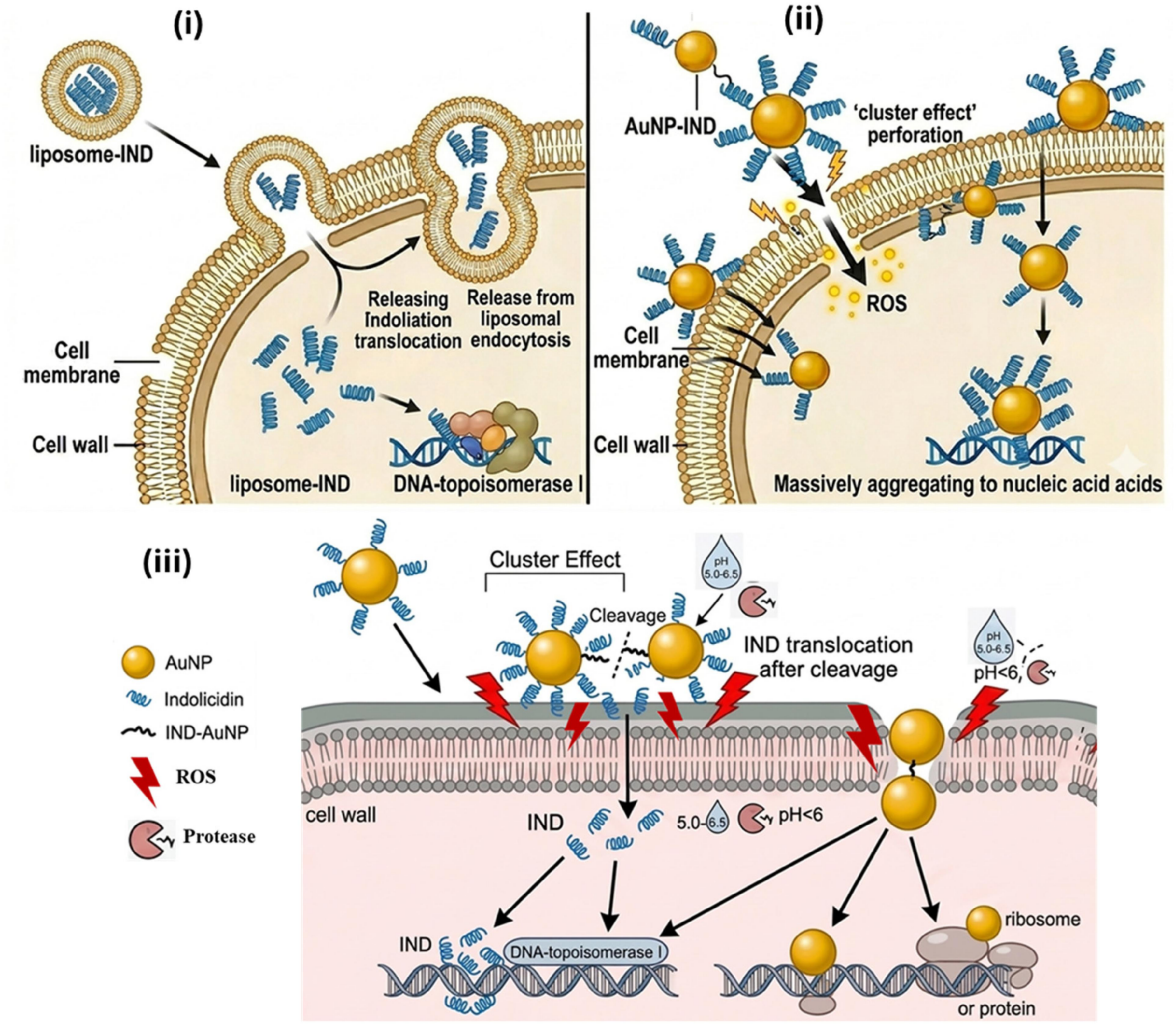
Mechanistic paradigms of indolicidin (IND) delivery. **i**. **(a)** Physical Encapsulation: Protects Ind within a degradable matrix, allowing for the release of the free peptide to execute its canonical biphasic mechanism and intracellular DNA targeting. **ii**. Non-Cleavable Conjugation: Permanent tethering restricts conformational freedom, inducing a “cluster effect” that shifts activity from intracellular targeting toward localized membrane disruption and Mg^2+^, Ca^2+^ displacement. **iii**. Stimuli-Responsive Conjugation: Employs cleavable linkers (e.g., pH or enzymatic) for programmed release at the infection site, facilitating synergistic dual-action (created with AI).

Upon carrier dissociation in the target microenvironment, Ind is released in its free, conformationally flexible state (Figure 3I). Crucially, the kinetics of release from the degradable matrix must be strictly matched to the infection site's enzymatic activity; premature release in the bloodstream results in systemic toxicity, while delayed release may permit bacterial adaptation (56). In this encapsulated scenario, the peptide is fully capable of executing its canonical biphasic mechanism, transient membrane permeabilization followed by intracellular topoisomerase I inhibition, upon liberation at the infection site (182). This preservation of canonical dual-mechanism activity is the defining pharmacodynamic advantage of encapsulated systems over covalently conjugated ones, and it is directly responsible for the superior systemic safety profiles documented for liposomal Ind (57). Nonetheless, it concurrently imposes rigorous requirements on the engineering of release kinetics that have yet to be fulfilled in a clinically validated formulation of Ind.

### 3.2 Non-cleavable covalent conjugation

From a thermodynamic perspective, effective membrane translocation by free Ind requires conformational flexibility to progressively insert and facilitate lipid flip-flop across the bilayer leaflets. Molecular dynamics simulations reveal that this process occurs with relatively low minimal free-energy barriers, estimated at approximately −15 to −20 kJ.mol^−1^ (depending on membrane composition and simulation force field) in DMPC bilayers, driven predominantly by the desolvation of tryptophan side chains, the burial of the hydrophobic wedge, and sustained salt-bridge interactions between the peptide's cationic residues and lipid phosphates (18, 58, 59). These favorable energetic dynamics are dramatically perturbed upon covalent surface conjugation (60, 61, 194). When Ind is permanently immobilized via stable chemistries such as thioether linkages, its amphipathic wedge-like conformation is severely constrained. Quantitative biophysical studies have demonstrated that the rotational freedom of surface-tethered antimicrobial peptides is substantially reduced compared to the free peptide, directly preventing the execution of the transient lipidic defects required for membrane translocation (30, 59, 61–64).

Ultimately, this restricted conformational freedom fundamentally alters the biophysics of Ind by shifting its action from a diffusion-limited intracellular targeting model to a highly localized, density-dependent membrane disruption model known as the “cluster effect.” When multiple Ind molecules are co-immobilized at high surface densities, the cumulative positive charge creates an electrostatic field that selectively attracts bacterial membranes over mammalian cells. Furthermore, the high local density of tryptophan residues generates a hydrophobic patch that synergistically disrupts the outer membrane of Gram-negative bacteria by intercalating between the acyl chains of lipopolysaccharide (LPS) molecules, displacing the Mg^2+^ and Ca^2+^ ions that normally stabilize LPS structural integrity (1, 55). While this permanent restriction limits traditional intracellular targeting, severe membrane depolarization induced by the cationic cluster can trigger uptake of the intact nanoparticle–peptide complex, inducing toxicity through generalized physical damage and oxidative stress rather than selective molecular binding (Figures 3ii).

### 3.3 Conjugation via cleavable ligation

Alternatively, anchoring Ind via stimulus-responsive spacers, such as pH-sensitive or enzyme-cleavable bonds, allows for a dual-action strategy (194). This approach represents the most mechanistically advanced nanocarrier strategy for Ind, as it could theoretically preserve the peptide's complete canonical dual mechanism while also incorporating the carrier's own independent antimicrobial activity. However, it also involves the highest level of formulation complexity and, importantly, currently has the least Ind-specific experimental validation among the three modalities discussed here. This approach is highly compatible with targeted delivery platforms, where the nanocarrier can be designed to respond to specific biomarkers at the infection site, releasing the peptide in a programmed manner (Figures 3iii). Upon linker cleavage, the dissociated nanoparticle component can independently simultaneously be employed for biosensing or localized generation of reactive oxygen species (ROS) (7).

The rational design of cleavable linkers requires careful selection of the chemical moiety: hydrazone bonds are cleaved at pH 5.0–6.5 (characteristic of endo-lysosomal and infectious microenvironments), disulfide bonds are reduced by glutathione (present at millimolar concentrations intracellularly vs. micromolar extracellularly), and protease-sensitive peptide linkers (e.g., Val-Cit-PABC) are cleaved by cathepsins B and D overexpressed at infection and tumor sites (65, 66). Following nanocarrier-mediated membrane penetration, the linker is cleaved in the periplasm or within the cytosol, releasing Ind to execute its inhibition of topoisomerase I. Concurrently, the dissociated nanoparticle can exert an independent line of attack, such as the localized generation of ROS, resulting in a synergistic lethal effect where the action of both components is additive and thereby prevents bacterial adaptation, See Figures 3iii (67, 200).

Understanding this mechanistic bifurcation is essential for the rational design of targeted therapies, as it translates to distinct clinical applications: surface-conjugated Ind-nanoparticles (bound state) are optimal for topical wound care and catheter coatings where persistent bactericidal surface activity is required, while encapsulated-Ind systems (free state) are preferable for treating deep-seated or systemic infections where intracellular bacterial pathogens must be targeted (3, 45, 68). Notably, however, direct comparative studies systematically evaluating both modes of action under equivalent experimental conditions remain scarce; rigorous head-to-head investigations are needed to validate and refine this mechanistic framework.

Indolicidin acts primarily after release from the carrier in encapsulation and cleavable-linker systems, where it retains its full canonical mechanism. In contrast, under non-cleavable covalent conjugation, Ind acts exclusively while bound, sacrificing intracellular topoisomerase I inhibition for enhanced surface-level membrane disruption. This distinction is not merely academic, it dictates clinical application: surface-conjugated systems are optimal for topical biofilm eradication, while encapsulated systems are required for systemic intracellular infections. Importantly, however, no studies to date have directly compared these mechanisms under the same experimental conditions using Ind itself rather than related AMPs. Side-by-side evaluations of the three conjugation modalities, performed with standardized bacterial strains, infection models, and outcome measures, remain a major gap in the literature and should be considered a key priority for future research.

## 4 Inorganic platforms: amplifying membrane disruption and ROS synergy

Inorganic platforms, such as gold nanoparticles (AuNPs), silver nanoparticles (AgNPs), and quantum dots (QDs), carbon nanotubes (CNTs), graphene oxide (GO), metal-oxide nanoparticles (TiO_2_, ZnO), and mesoporous silica nanoparticles (MSNPs), act as rigid scaffolds that fundamentally alter the pharmacodynamics of Ind. Unlike organic carriers that primarily serve as passive vehicles, inorganic nanoparticles are active participants in the bactericidal process, contributing their own intrinsic antimicrobial mechanisms that operate in concert with the conjugated peptide (191). By presenting the peptide in a multivalent fashion, these platforms induce a “cluster effect” that drastically increases local positive charge density, facilitating potent bacterial membrane depolarization. Specifically, the accumulation of cationic Ind molecules disrupts the outer membrane of Gram-negative bacteria by displacing divalent cations (Mg^2+^ and Ca^2+^) that normally stabilize the lipopolysaccharide (LPS) layer, (60, 69, 201). Kumar et al. (70) further established that the selectivity of this interaction for bacterial over mammalian membranes is enhanced by the high content of phosphatidylethanolamine (PE), anionic lipids such as phosphatidylglycerol (PG) and cardiolipin in bacterial membranes relative to mammalian cells, which present predominantly phosphatidylcholine (PC) and sphingomyelin, both of which are less electrostatically responsive to Ind's cationic cluster (70, 162).

Beyond structural damage, the mechanistic synergy in these systems is enhanced by the intrinsic ability of inorganic cores (especially AgNPs and QDs) to generate reactive oxygen species (ROS). The primary mechanisms of ROS generation differ between platforms: AgNPs release Ag⁺ ions that disrupt the bacterial electron transport chain, leading to superoxide (O_2_.^−^) and hydrogen peroxide accumulation; QDs composed of CdSe or CdTe generate singlet oxygen (^1^O_2_) and superoxide upon photoexcitation through semiconductor band-gap transitions; while AuNPs, being chemically inert, rely primarily on photothermal ROS generation upon near-infrared (NIR) irradiation rather than spontaneous chemical reactivity (1, 71). The localized lipid peroxidation induced by the nanoparticle reduces the mechanical resistance of the bacterial membrane, allowing the surface-bound Ind cluster to efficiently insert its tryptophan-rich domains and cause catastrophic depolarization (55, 72–74). This dual attack, physical disruption by the peptide and oxidative stress by the core, effectively prevents the selection of resistant strains, showing superior efficacy even against fungal pathogens such as *C. albicans* (72). While the synergistic effect is well-documented, the causal direction remains unclear: nanoparticle-generated ROS may oxidize membrane lipids, reduce mechanical resistance and lowering the threshold for Ind insertion. Alternatively, Ind-mediated membrane permeabilization may enhance ROS penetration. Direct mechanistic studies using ROS scavengers and lipid oxidation assays are needed.

Gold nanoparticles (AuNPs) represent arguably the most extensively characterized platform for Ind conjugation, owing to the specificity and stability of thiol-gold chemistry. Rahimi et al. (41) conjugated Ind to AuNPs via thiol chemistry, engineering a cysteine-terminated peptide to ensure controlled orientation. These Ind-AuNP conjugates inhibited fluconazole-resistant *C. albicans* with 99.9% viability reduction and were associated with downregulation of ERG11, encoding the lanosterol 14*α*-demethylase that constitutes the primary target of azole antifungals, providing molecular evidence that Ind-AuNPs bypass the ergosterol biosynthesis pathway, consistent with a membrane-surface disruption mechanism independent of azole resistance (41). Similarly, de Alteriis et al. (45) demonstrated that AuNPs functionalized with Ind via thiol chemistry achieved a 4- to 8-fold improvement against fluconazole-resistant *C. albicans* biofilms strictly via targeted surface-level interactions (45). In a complementary study, de Alteriis et al. (68) reported an early genotoxicity assessment of Ind-functionalized AuNPs using *Saccharomyces cerevisiae*, identifying reduced DNA-damaging effects of the Ind-AuNP conjugate compared to bare cationic gold particles, a finding that positions Ind surface coating as a partial biosafety strategy warranting further ecotoxicological validation (68).

A notable evidence gap in the AuNP-Ind literature is its exclusive focus on *Candida spp*. and immunomodulation endpoints; no published study has systematically evaluated Ind-AuNP conjugates against priority WHO-listed bacterial pathogens such as carbapenem-resistant *Acinetobacter baumannii* or extended-spectrum *β*-lactamase (ESBL)-producing Enterobacteriaceae using clinically relevant infection models. This represents a critical evidence gap that must be addressed before AuNP-Ind systems can be proposed for clinical development against bacterial AMR (190).

Approaching AuNP functionalization from an immunological perspective, Sur et al. (75) utilized carbodiimide (EDC/NHS) chemistry to covalently attach Ind to both AuNPs and carboxylated carbon nanotubes (CNTs), demonstrating that Ind-AuNP conjugates enhanced the immune-stimulatory activity of indolicidin in THP-1 macrophages at a 1,000-fold lower effective dose (0.02 µg.mL^−1^) compared to the free peptide (20 µg.mL^−1^), conferring superior protection against *Salmonella typhimurium* infection *in vitro* (75). In a parallel approach, Pradhan et al. (76) expanded this work by performing a comparative immunomodulatory analysis of Ind and LL-37 conjugated to CNTs, confirming that Ind-CNT activates macrophage signaling via the TNFRSF1A/NF*κ*B/c-JUN pathway and provides significant protection of THP-1 cells against *S. typhimurium* at subtherapeutic peptide concentrations (76). The mechanism of biofilm penetration by Ind-AuNPs is particularly noteworthy: nanoparticles in the 5–15 nm size range diffuse through the extracellular polymeric substance (EPS) matrix of biofilms via charge-mediated interactions, a property that free Ind cannot replicate due to its rapid adsorption onto the outer biofilm layers. Furthermore, Ind-AuNPs exhibit markedly enhanced photostability compared to the free peptide, rendering them compatible with photodynamic antimicrobial chemotherapy (PACT) protocols (45, 65). The multivalent surface display on AuNPs also increases the avidity of Ind-membrane interactions by several orders of magnitude relative to monomeric free Ind, since membrane binding in the “cluster” format is not diffusion-limited but rather depends on nanoparticle-membrane collision frequency, which is governed by nanoparticle diffusion coefficients in the bacterial periplasm (77, 175).

Silver nanoparticles (AgNPs) present a mechanistically richer but toxicologically more complex profile. To harness their potential, various conjugation strategies have been developed. For instance, Zharkova et al. (74) incorporated indolicidin into a gelatin-based coating on AgNPs. The resulting AgNP-indolicidin conjugate retained strong antibacterial activity against drug-resistant strains, including *E. coli*, *Klebsiella spp*., *Pseudomona aeruginosa*, and *Staphylococcus aureus* (74). Similarly, Zannella et al. (78) described the formation of a colloidal AgNP-Ind conjugate using hydrazine monohydrate as a reducing agent, reporting potent activity against *E. coli*, *P. aeruginosa* and *S. aureus* in the context of oral pathologies (78). To further improve the translational potential of these metallic platforms, Wan et al. (79) demonstrated that chitosan coating of AgNPs substantially enhanced colloidal selectivity and mammalian biocompatibility while maintaining potent antimicrobial activity, establishing cationic polymer coating as an adjunctive strategy to Ind functionalization (79).

The translational pathway for Ind-AgNP systems presents an additional and often underestimated challenge. Unlike AuNPs, silver nanoparticles are not chemically inert, and their dissolution kinetics in physiological fluids are highly dependent on formulation variables such as particle size, surface coating, and ionic strength. Consequently, the Ag^+^ release profile, which is a major determinant of both antimicrobial activity and toxicity, is likely to differ markedly between the controlled conditions used in published Ind-AgNP studies and the protein-rich microenvironments encountered at *in vivo* infection sites. This variability represents a substantial barrier to preclinical-to-clinical translation and has not yet been systematically evaluated for Ind-AgNP formulations.

The mechanistic basis for the enhanced synergistic activity of Ind-AgNP conjugates against MDR pathogens, including *A. baumannii* relates to a two-pronged synergistic two-pronged attack. First, the capacity of Ind to permeabilize the outer membrane facilitates Ag^+^ ion penetration into the periplasm. Once inside, these ions directly inhibit bacterial respiratory enzymes, including NADH dehydrogenase and succinate dehydrogenase, thereby collapsing the proton motive force (80, 81). This is particularly effective against multidrug-resistant strains that rely on outer membrane impermeability as a primary resistance mechanism. However, a critical design parameter for these Ind-AgNP systems is the ratio of Ind surface coverage to exposed Ag surface area: insufficient Ind coverage leaves metallic silver exposed to plasma proteins, triggering rapid opsonization and phagocytic clearance, while excessive Ind loading may passivate the silver surface and severely reduce essential Ag^+^ ion release kinetics. When optimized, this surface-immobilized Ind layer not only drives targeted antimicrobial action but also provides a passivating effect that improves the overall ecotoxicological and biosafety profile, as demonstrated in *Daphnia magna* and plant seed models (82).

Expanding the repertoire of CNT-based Ind conjugates, Pradhan et al. (76) conducted a comparative immunomodulatory analysis of Ind and LL-37 conjugated to CNTs, confirming that Ind-CNT activates macrophage signaling via the TNFRSF1A/NF*κ*B/c-JUN pathway and provides significant protection of THP-1 cells against at subtherapeutic peptide concentrations (76). The remarkable potency enhancement observed with CNT-Ind conjugates has been mechanistically attributed to two complementary factors: (i) the high aspect ratio of CNTs facilitates physical membrane penetration, acting as a “nano-needle” that creates focal entry points for Ind clusters; and (ii) the π-system of CNTs engages in strong π-π stacking interactions with the indole rings of Ind's tryptophan residues, maintaining optimal peptide orientation for membrane insertion while providing thermodynamically stable conjugation that resists competitive displacement by serum proteins (76, 83). Galdiero et al. (72) further demonstrated that surface functionalization of CNTs significantly increased the local multivalent density of Ind, amplifying membrane disruption through the cluster effect (72).

The non-covalent supramolecular loading onto graphene oxide (GO) or PEGylated nanotubes has emerged as an effective alternative. These platforms utilize robust π-π stacking and electrostatic interactions to achieve high loading capacities while permitting stimuli-driven release in the infectious microenvironment (59, 84, 85). Specifically, GO surfaces are particularly attractive: their oxygen-containing functional groups (carboxyl, hydroxyl, and epoxide) confer a negative surface charge at physiological pH, enabling electrostatic immobilization of cationic Ind with loading efficiencies of typically 60%–85% by mass without requiring chemical modification of the peptide. The acidic microenvironment of infection sites (pH 5.5–6.5) partially protonates GO carboxylate groups, reducing electrostatic interactions and facilitating pH-responsive Ind release, an elegant mechanism that exploits the pathological environment as a biochemical trigger without requiring covalent linker engineering (84, 86). Farzanegan et al. (87) validated this approach by formulating a novel GO-indolicidin nanocomposite that preserved the peptide's structural integrity and intracellular activity, exhibiting potent *in vivo* antifungal activity against disseminated candidiasis with a minimized toxicity profile (87). Furthermore, exploring non-traditional metal oxides as alternative non-covalent carriers, Masoumi et al. (88) reported the successful loading of Ind onto TiO_2_ and ZnO nanoparticles. This strategy exploits their intrinsic photocatalytic ROS generation to synergize with Ind's membrane-disturbing properties (88, 89).

The quantum dot (QD) platform offers the unique advantage of simultaneous fluorescent diagnostic imaging and bactericidal therapy (theranostics); however, it is encumbered by the intrinsic cytotoxicity of heavy-metal semiconductor cores. Galdiero et al. (72) formulated theranostic conjugates by covalently linking Ind to amine-functionalized QDs via EDC/NHS activation, finding that functionalization significantly improved antibacterial activity while mitigating the acute mortality typically induced by bare QDs. However, in a critical complementary study, Maselli et al. (90) subsequently characterized the long-term ecological impact of these QD-Ind conjugates on *D. magna,* revealing that despite an improved acute safety profile, the conjugates induced multigenerational reproductive alterations and dose-dependent DNA damage (188, 189). This persistent genotoxicity reflects a broader ecotoxicological challenge associated with metallic and semiconductor nanosystems, underscoring the urgent need for safer, biocompatible platforms and is discussed in greater detail in **Section 6** (71, 91).

To overcome the fundamental diffusion limitations of passive nanoparticles in penetrating the dense EPS of mature biofilms, active microrobotics have emerged as a disruptive technological paradigm. Milosavljevic et al. (92) engineered self-propelled multifunctional microrobots functionalized with Ind via nanoarchitectonics, demonstrating the unique capacity of these microswimmers to autonomously navigate toward bacterial cells, mechanically penetrate established MRSA biofilms, and deliver Ind directly to the biofilm interior. This localized delivery successfully restored the peptide's topoisomerase I inhibitory function *in situ*, exhibiting excellent selectivity and superior bactericidal activity compared to free Ind (92). The integration of Ind into microrobotic architectures thus represents a conceptually distinct third category of nanoconjugation, beyond mere surface tethering and passive encapsulation, that synergistically combines mechanical physical disruption with targeted molecular lethality. Jancik-Prochazkova et al. (93) recently validated the *in vivo* efficacy of this active delivery paradigm by eradicating antibiotic-resistant *S. aureus* skin colonies (93). Looking ahead, biohybrid platforms incorporating broad-spectrum AMPs such as Ind represent a high-priority research direction for combating biofilm-associated infections at catheter surfaces, orthopedic implant sites, and surgically inaccessible locations (94, 202).

A critical mechanistic question concerns the causal relationship between nanoparticle-generated ROS and Ind's antimicrobial activity. Three non-mutually exclusive mechanisms may operate: (i) ROS-mediated lipid peroxidation reduces membrane mechanical resistance, lowering the energetic barrier for Ind insertion (72, 95); (ii) Ind-induced membrane permeabilization enhances ROS penetration to intracellular targets (96); or (iii) both agents act independently on parallel lethal pathways (17). While the synergistic effect is well-documented, direct causal evidence remains limited. To our knowledge, no study has performed ROS scavenger experiments (e.g., using N-acetylcysteine or vitamin C) to determine whether ROS neutralization abolishes synergy with Ind-nanoparticle conjugates (78, 97, 98). We therefore recommend this as a priority for future mechanistic investigations. The available evidence, however, is consistent with a bidirectional amplification model: Ind's membrane disruption facilitates ROS entry, while ROS-induced damage sensitizes the membrane to further peptide insertion (96, 99, 203).

Despite this promising *in vivo* proof of concept, Ind delivery via microrobots still faces important translational challenges. Manufacturing these systems under Good Manufacturing Practice (GMP) conditions remains technically unfeasible, the long-term fate of their inorganic components in tissues is still unclear, and their ability to navigate complex three-dimensional tissue environments has not yet been demonstrated. Consequently, Ind-based microrobots should currently be considered a promising but still early-stage platform that requires substantial engineering and regulatory development before clinical applicability can be realistically assessed.

Mesoporous silica nanoparticles (MSNPs) have emerged as an advanced delivery platform that addresses the inherent toxicity of heavy-metal cores. The well-defined and tunable pore structure (2–10 nm) of MSNPs, combined with a silanol-rich surface amenable to electrostatic interaction with cationic payloads, confers substantial potential for loading cationic AMPs such as Ind (+4 net charge) via electrostatic attraction, and for pH-sensitive cargo release driven by the partial protonation of surface silanols in the acidic infectious microenvironment (pH 5.5–6.5), a stimulus-free release mechanism of particular clinical relevance, as it exploits the pathological microenvironment itself as the biochemical trigger without requiring exogenous energy input or covalent linker engineering (65, 83, 187).

Despite these theoretical advantages, Ind-specific MSNPs formulations remain largely unexplored; no published study has yet evaluated a platform where Ind serves as the primary encapsulated payload. Currently, the closest precedent is a dual-payload system designed by Alharthi et al. (100), where small-molecule sortase A inhibitors (SrtAIs) were loaded into the interior pores of unmodified (MCM-41) or phosphonate-functionalized (MCM-41-PO_3_^−^) MSNPs, while Ind was electrostatically deposited as an outer surface coating. This hybrid architecture demonstrated broad-spectrum efficacy against both Gram-positive (MSSA, MRSA) and Gram-negative (*E. coli*, and *P. aeruginosa*) pathogens. Notably, the phosphonate-modified matrices yielded superior MICs and marked synergy (FICI < 0.5) when combined with Ind. Mechanistically, this synergy arises because MSNP-mediated delivery of SrtAIs disrupts virulence factor anchoring and weakens the Gram-positive cell wall, lowering the activation threshold required for surface-bound Ind to execute its membrane-permeabilizing activity (100, 170).

While modified MSNPs variants have well-established capabilities for stimuli-responsive antibiofilm therapy (83), these paradigms have rarely been extended to AMP core payloads. Consequently, critical translational parameters, such as the optimal Ind-to-MSNPs loading kinetics, pore size compatibility with the peptide's specific wedge-like topology, pH-dependent desorption profiles, and subsequent *in vivo* therapeutic outcomes, remain completely uncharacterized. Overcoming these development gaps through systematic formulation optimization and pharmacokinetic profiling represents a high-priority objective for next-generation AMP nanomedicines.

From a regulatory standpoint, silicon dioxide carries GRAS classification by the FDA as a food additive; however, this designation does not extend automatically to MSNPs administered as intravenous nanomedicines, and independent preclinical safety evaluation, including biodistribution, chronic toxicity, and silica dissolution kinetics in biological fluids, is a mandatory prerequisite for any pre-IND submission of an Ind-MSNP formulation. Nevertheless, this silica-based chemistry positions MSNPs as a more tractable regulatory starting point relative to heavy-metal or semiconductor nanocarrier cores, provided that the Ind-specific efficacy data required to justify clinical development are generated. Taken together, MSNPs occupy a theoretically favorable position among inorganic delivery platforms, combining an FDA-recognized chemical scaffold, tunable stimulus-responsive functionality, and a mechanistically grounded rationale for Ind loading, but this theoretical advantage can only be actualized through the formulation-specific experimental validation that the current literature conspicuously lacks.

Taken together, the inorganic platform literature for Ind delivery presents a pattern of impressive but narrow proof-of-concept data: potency gains are frequently demonstrated within a single pathogen-platform pair, under standardized *in vitro* conditions, without systematic investigation of mechanism confirmation, pharmacokinetic behavior, or safety in relevant *in vivo* models. The field requires multi-platform, multi-pathogen comparative studies using Ind specifically, with and without the nanocarrier, across both membrane-disruption and intracellular endpoints, to determine whether the efficacy gains attributed to inorganic conjugation are mechanistically attributable to the cluster effect, to ROS synergy, to enhanced biofilm penetration, or to some combination thereof. This mechanistic disambiguation is not a merely academic exercise; it is essential for informed clinical indication selection and for identifying which resistance mechanisms each platform-Ind combination can and cannot overcome.

## 5 Organic and responsive carriers: controlled release and biocompatibility

Unlike covalently functionalized inorganic platforms, organic carriers, including liposomes, polymeric nanoparticles, and hydrogels, are strategically designed to preserve Ind's canonical biphasic mechanism of action by sequestering the peptide within a biodegradable matrix and releasing it in a free, conformationally flexible state at the target site (166, 173, 187, 193). These architectures also act as molecular shields that mitigate Ind's intrinsic toxicity toward host cells by preventing direct, non-specific interactions between its amphipathic domains and zwitterionic mammalian cell membranes. This masking capability represents a critical pharmacological advantage, given that the therapeutic index of free Ind is critically narrow when deployed against highly resistant pathogens (97, 176).

Liposomal systems represent the most mature lipid-based platform for Ind delivery, mitigating the pharmacokinetic barriers and cytotoxicity of free AMPs (97, 101). Ahmad et al. (57) developed palmitoyloleoylphosphatidylcholine (POPC)-based liposomes to encapsulate Ind, achieving near 100% encapsulation efficiency and a remarkable expansion of the therapeutic index: the liposomal formulation reduced non-specific toxicity, increased IC_50_ 16-fold in CHO/K1 cells, and significantly attenuated hemolysis. In murine models, encapsulation elevated the maximum tolerated dose, enabling safe systemic administration at 40 mg.kg^−1^, whereas free Ind exhibited lethality at just 0.9 mg.kg^−1^. Consequently, this formulation a 30% long-term survival rate and a complete microbiological lung cure in mice with systemic aspergillosis, a therapeutic outcome entirely unattainable with the free peptide (57).

While Ind-specific plasma pharmacokinetic (PK) parameters from liposomal formulations remain systematically underreported, an omission that underscores a critical gap in the translational literature, the observed toxicological mitigation strongly implies improved systemic parameters (165, 195). By analogy with established stealth liposomal technologies, bilayer encapsulation and surface PEGylation are theoretically expected to shield the structurally vulnerable peptide from intravascular protease degradation and retard renal clearance, shifting the profile of Ind from a rapidly cleared molecule to a sustained-release therapeutic (167).

Crucially, liposomal encapsulation is not a universally beneficial intervention; performance depends strictly on lipid architecture. Ron-Doitch et al. (48) conducted a comparative study encapsulating both LL-37 and Ind separately in PEGylated distearoylphosphatidylcholine (DSPC) liposomes against herpes simplex virus type 1 (HSV-1), revealing a critically instructive asymmetry. While liposomal LL-37 exhibited reduced host toxicity and enhanced antiviral efficacy, liposomal Ind demonstrated significantly greater host cell toxicity than the free peptide (CC_50_ of 19.1 µM vs. 47.7 µM, respectively; *p* < 0.05), with no improvement in antiviral potency. The mechanistic basis for this divergence stems from Ind's disordered, wedge-like topology. This conformation is fundamentally incompatible with the highly ordered lamellar organization of DSPC bilayers, forcing the peptide into a shallow, surface-level association that generates highly membranolytic, non-lamellar lipid-peptide phases on the vesicle exterior. Conversely, LL-37's structured *α*-helical amphipathicity permits deeper bilayer integration and stable encapsulation within DSPC membranes, explaining its superior performance in the same lipid system (8).

This structural incompatibility demonstrates that direct cross-study comparisons between foundational works, such as the fluid-phase POPC matrices used by Ahmad et al. (57) and the gel-phase DSPC networks used by Ron-Doitch et al. (48), are inherently confounded by unstandardized lipid-to-peptide molar ratios and divergent experimental endpoints. Consequently, multi-parametric optimization matrices that systematically vary bilayer fluidity, phase transition temperatures, and surface-stabilization coatings remain an urgent prerequisite to ensure liposomal vectors predictably diminish, rather than exacerbate, Ind-mediated host cytotoxicity (165).

The physical interaction between Ind and liposomal membranes is mechanistically complex. At sub-lytic concentrations, Ind partitions into the outer leaflet of the liposomal bilayer, generating curvature stress and increasing membrane permeability in a dose-dependent manner without vesicular rupture. This behavior mirrors its interaction with bacterial membranes, explaining why liposomes must be engineered with membrane-stabilizing lipids to prevent premature peptide leakage during storage and systemic circulation (56). The surface charge of liposomal systems dictates their *in vivo* fate: cationic liposomes enhance bacterial binding but suffer from rapid opsonization, whereas PEGylated neutral liposomes achieve prolonged circulation but require active endosomal escape mechanisms (167). To facilitate intracellular delivery, advanced formulations incorporate fusogenic lipids (e.g., DOPE) that adopt an inverted hexagonal phase upon interaction with bacterial or endosomal membranes under acidic conditions, physically ejecting Ind directly into the bacterial cytoplasm and restoring topoisomerase I inhibitory activity, a mechanism impossible to achieve with stably anchored covalent conjugates. Such strategies are paramount for eradicating deep-seated infections within complex polymicrobial architectures such as mature oral biofilms (102). A further PK consideration that is critical for Ind liposome design is the route-dependent release profile: inhalation-administered liposomal oseltamivir phosphate dry powders demonstrated AUC (the area under the plasma concentration–time curve of a drug after administration) values 1.14-fold higher than oral solution alongside a significantly delayed T_max_ (1.55 h vs. 0.25 h), confirming that the pulmonary route dramatically extends local drug residence time, a principle directly applicable to Ind liposomal aerosols for the management of respiratory infections (103).

Liquid-crystalline cubosomes have emerged as structurally sophisticated vehicles for lipophilic and amphipathic payload delivery, offering unique advantages and distinct physicochemical constraints when applied to Ind. Meikle et al. (86) demonstrated that the internal bicontinuous cubic structure forms a complex interconnected network of water channels capable of accommodating cationic AMPs, including Ind, within an electrostatically protected environment. Notably, phytantriol-based cubosomes loaded with Ind achieved MICs of 8 μg.mL^−1^ against *S. aureus* and 4 μg.mL^−1^ against *B. cereus*, representing at least a 2-fold improvement in antimicrobial potency relative to the unencapsulated free peptide under these formulation conditions (86).

However, this platform performance is highly lipid-dependent rather than a class-level attribute. Lakic et al. (104) conducted a head-to-head comparative encapsulation study using monoolein (MO)-based cubosomes loaded with Ind and its shorter synthetic analog, Priscilicidin (Prs), to interrogate how peptide structural identity governs cubosomal phase behavior. Cryo-TEM analysis demonstrated that Ind destabilized the cubic arrangement at a loading as low as 1 mol%, yielding predominantly unstructured vesicles, whereas Prs preserved the ordered cubic lattice under identical conditions. This structural collapse is attributable to Ind's greater curvature stress arising from its disordered wedge-like topology and elevated cationic charge density relative to the more compact Prs sequence (104). While supplementing the formulation with 150 mM NaCl during preparation electrostatically screened Ind's cationic charge, raising encapsulation efficiency from 32% to 96% and preventing structural collapse, these NaCl-stabilized MO cubosomes showed lower antibacterial efficacy against *E. coli* than the free peptide, with activity declining further at 2 mol% loading (104).

This therapeutic decline exposes a critical biophysical trade-off: the ionic screening required to preserve the cubic lattice simultaneously dampens the electrostatic driving forces necessary for Ind to interact productively with bacterial target membranes. Furthermore, restricted peptide mobility within the dense lipidic matrix retards its outward diffusion. Because Ind is an intrinsically high-permeability molecule that traverses bacterial envelopes rapidly as a free monomer, this matrix encapsulation conflicts with the classical “diffusion-to-capture” model. This model posits that nanocarrier encapsulation yields functional benefits primarily for payloads with poor intrinsic membrane permeation rates; for Ind, immobilization within a stable cubosomal phase slows rather than accelerates transport to its sub-cellular targets (86, 104).

Consequently, the translational utility of standard cubosomes for Ind is structurally constrained. The platform retains genuine viability only in specialized niches where phase disruption can be mitigated, such as low-loading topical dermal applications or hybrid lipid-polymer cubosomal architectures where a polymeric scaffold artificially stabilizes the cubic phase against peptide-induced steric stress. This cubosomal case study underscores a recurring paradox in AMP nanomedicine: the very structural properties that endow Ind with high pharmacological potency, namely its dense cationic charge, aromatic bulk, and high membrane affinity, are precisely the elements that trigger matrix incompatibility. Therefore, future development programs must prioritize pre-formulation compatibility matrices and phase behavior screenings as an absolute prerequisite before advancing to *in vivo* efficacy modeling (105, 184).

Polymeric nanoparticles (PNPs), particularly those composed of PLGA, chitosan, and poly(*ε*-caprolactone), offer complementary advantages to lipid-based systems through highly customizable, biodegradable matrices (176, 181, 185). PLGA-based PNPs encapsulating AMPs such as Ind demonstrate pH-triggered release profiles: acid-catalyzed ester hydrolysis of PLGA within the endosomal environment (pH 5.0–5.8) accelerates polymer degradation, releasing the AMP directly within intracellular compartments, a mechanism particularly relevant for treating infections caused by facultative intracellular pathogens such as *S. enterica* and *Listeria monocytogenes* (106–108). Exploiting natural biopolymers, Rata et al. (109) demonstrated that chitosan-based systems utilize an additional selectivity mechanism: the cationic charge of chitosan at acidic pH synergizes with Ind's intrinsic positive charge, creating a “charge amplification” effect that dramatically enhances bactericidal membrane interactions in the acidic infectious microenvironment while maintaining near-neutral charge at physiological pH to minimize host cell toxicity (109). Cadinoiu et al. (110) recently engineered chitosan–Ind hybrid nanoparticles with optimized surface chemistry, confirming concentration-dependent biofilm inhibition against MRSA with a favorable cytotoxicity profile in human keratinocyte lines, strongly supporting their suitability for topical wound applications (110). The inherent complementary antimicrobial activity of the chitosan backbone provides an additional layer of antibacterial activity synergistic lethality alongside Ind and the nanostructure itself (79).

A critical limitation common to all polymeric Ind nanoparticle studies reviewed here, including the chitosan systems of Cadinoiu et al. (110) and Rata et al. (109), is the lack of standardized *in vivo* pharmacokinetic data (t_1/2_, AUC, C_max_, tissue distribution) for Ind as the encapsulated agent (185). Importantly, this issue is not restricted to polymeric formulations but is consistently observed across the broader Ind nanocarrier literature, representing one of the field's main translational gaps. Without pharmacokinetic characterization, it remains unclear whether the improved *in vitro* MIC values reported for these systems can translate into meaningful *in vivo* efficacy at clinically achievable tissue concentrations. For this reason, future *in vivo* studies should incorporate pharmacokinetic evaluation as a core component of therapeutic validation.

The incorporation of AMPs into hydrogels has emerged as a highly effective strategy for localized topical therapies and wound management (184, 193). Recktenwald et al. (111) demonstrated that Ind immobilized within polyethylene glycol (PEG)-based hydrogel matrices at its MIC (43.8 μM) effectively prevented MRSA adhesion and biofilm formation (111). For spatiotemporal control over peptide release, temperature-responsive poly(N-isopropylacrylamide) (PNIPAM) hydrogels undergo an entropy-driven sol–gel transition upon warming to body temperature (>32° C), physically entrapping Ind within the collapsed polymer network and subsequently releasing it in response to localized hypothermia or enzymatic degradation of the backbone (112, 113, 187). Haider et al. (114) and Hatae et al. (101) further developed magnetic nanoparticle-loaded PNIPAM hydrogels incorporating Fe₃O₄ NPs, enabling on-demand, remote-controlled Ind release triggered by alternating magnetic field (AMF)-induced localized hyperthermia (174).

Critically, the PK advantages demonstrated across all these organic platforms must be evaluated against two translational constraints that apply with equal force to Ind-based systems. First, the biomolecular corona problem: upon entry into human plasma, all lipid-based nanocarriers are immediately coated by a complex multilayered adlayer of plasma proteins, primarily albumin, fibronectin, immunoglobulins, and complement components, that fundamentally alters the nanoparticle's biological identity, replacing its engineered surface chemistry with a protein-defined interface that governs biodistribution, cellular uptake, and clearance rate (115, 116, 178). The corona composition is highly species-specific, making direct PK extrapolation from rodent models to human pharmacokinetics unreliable without species-specific corona characterization, a concern particularly acute for cationic Ind-loaded systems, whose positive surface charge is intrinsically opsonin-attractive. Second, manufacturing scalability: while liposomes benefit from established GMP-compliant processes and regulatory precedent, cubosomal systems present significant fabrication challenges arising from complex phase behavior, high internal viscosity, and the sensitivity of the bicontinuous cubic lattice to shear forces during scale-up, challenges compounded for Ind by the phase-disruption effects described above. Collectively, these organic and polymeric platforms represent the most clinically translatable category of Ind delivery systems, aligning with established FDA regulatory frameworks for polymer nanomedicines and offering a vastly superior safety profile for both systemic and topical administration (168, 169, 172, 182).

## 6 Beyond antimicrobial applications: Ind as a gene delivery excipient

Beyond its canonical bactericidal applications, the integration of Ind with polyethyleneimine (PEI) constitutes a conceptually distinct paradigm: repurposing the peptide as a multifunctional biomaterial for non-viral gene delivery. The pharmacological repertoire of AMPs extends far beyond membrane disruption, encompassing potent intracellular interactions such as high-affinity nucleic acid binding (23). Exploiting this capability, Hu et al. (117) pioneered the transition from unstable binary complexes to highly stable ternary polyplexes by self-assembling Ind with PEI and plasmid DNA. In this architecture, the cationic residues of Ind (arginine and lysine) synergistically condense nucleic acids, while its intrinsic membrane-permeabilizing activity efficiently facilitates endosomal escape, uniting two critical transfection mechanisms that typically require separate chemical entities (117).

The translational potential of PEI is historically bottlenecked by severe cellular cytotoxicity. Addressing this critical limitation, Hu et al. (118) demonstrated that engineering Ind into PEI-based polyplexes successfully neutralizes the polymer's inherent toxicity without compromising robust transfection efficacy for plasmid DNA, siRNA, and miRNA. In these formulations, the peptide acts as a steric and electrostatic “toxicity buffer” by displacing PEI from the outer polyplex surface, thereby mitigating damaging non-specific membrane interactions with host cells (118). Further refining this platform for clinical application, Tsai et al. (119) systematically evaluated the colloidal stability and gene condensation efficiency of PEI-Ind polyplexes across a range of nitrogen-to-phosphate (N/P) ratios, identifying optimal physicochemical parameters for *in vivo* applicability and firmly establishing Ind as a structural, dual-function excipient capable of advancing non-viral gene therapy vectors (119).

Further expanding the nucleic acid delivery applications of Ind beyond PEI-polyplex systems, Hu et al. (120) explored the use of indolicidin dimers as standalone vehiculization agents for oligodeoxynucleotides (ODNs). Two dimer configurations, designated LIC and CIL, differing in the orientation of peptide linkage, were designed to augment the charge density of monomeric Ind, thereby enhancing nucleic acid condensation capacity. Both dimers were evaluated as delivery vehicles for ODNs targeting tumor necrosis factor *α* (TNF-α), a pro-inflammatory cytokine of direct relevance to chronic infection and sepsis pathophysiology. The CIL dimer configuration demonstrated superior vehiculization capacity, achieving TNF-α expression silencing for over 14 h, a duration of suppression that positions CIL/ODN complexes as a potentially relevant platform for applications in gene silencing and immunomodulatory control of inflammatory infections (120). These results complement the PEI-polyplex work by confirming that Ind's nucleic acid-interacting capacity is intrinsic to its sequence and does not require polymeric scaffolding, a finding directly consistent with the PWWP motif's documented intercalation into B-type DNA duplexes described in Section 2. Taken together, the gene delivery literature positions Ind not merely as a membrane-active antimicrobial but as a multifunctional nucleic acid-binding scaffold whose delivery applications may ultimately extend beyond infection management into inflammatory disease and gene therapy, representing a translational frontier warranting dedicated investigation.

## 7 Genotoxicity and ecotoxicological analysis

Despite the potent efficacy demonstrated by the aforementioned inorganic systems, their clinical translation is heavily constrained by critical ecotoxicological and genotoxic concerns. These concerns are not peripheral considerations but rather fundamental regulatory prerequisites: both the FDA's 2022 guidance on nanomaterial-containing drug products and the EMA/CHMP guidelines mandate comprehensive genotoxicity characterization as a core component of the Ind-enabling safety package, alongside standard physicochemical and pharmacokinetic characterization (121, 171). The safety analysis of these nanomaterials must therefore extend beyond immediate cell viability to encompass long-term impacts on genomic integrity and biopersistence.

The most thoroughly documented example of this safety challenge concerns QDs covalently functionalized with Ind (QD-Ind). Acute toxicity tests on the aquatic indicator *Daphnia magna* showed improved safety and lower immediate mortality compared to unmodified QDs (72, 90). Strikingly, however, multigenerational exposure models spanning three consecutive generations revealed a cryptic toxicological scenario: despite low acute toxicity, chronic exposure to QD-Ind caused substantial DNA alterations and a marked multigenerational reproductive decline, including significant decreases in total egg count, broods per female, and overall body length (90, 91).

At the molecular level, these effects were correlated with upregulation of stress and detoxification genes (Dhb, CYP4, CYP314) and downregulation of the reproductive gene vitellogenin (*Vtg*). The reproductive decline is consistent with cadmium bioaccumulation kinetics documented for CdSe/ZnS QDs, wherein Cd^2+^ ions released during chronic degradation of surface coatings accumulate within reproductive tissues. Cadmium competitively displaces zinc from zinc-finger transcription factor binding sites and disrupts calcium-dependent embryonic signaling in invertebrates, providing a plausible mechanistic basis for the observed *Vtg* downregulation (90). This analysis exemplifies what may be termed a “toxicological paradox”: while peptide conjugation lowers the immediate acute toxicity of bare nanoparticles, the long-term biological accumulation and transgenerational genotoxicity of these inorganic carriers pose profound risks that must inform future nanosystem design.

Similarly, the intense oxidative stress characteristic of silver-based platforms may compromise mammalian epithelial cell viability if Ind surface coverage is not rigorously optimized to passivate the metal. Interactions between metallic cores and cellular components can induce chromosomal aberrations, underscoring the necessity of adopting biodegradable carrier architectures for applications where environmental discharge or prolonged systemic use is anticipated. A critical methodological caveat in genotoxicity evaluation is the “nanoparticle interference effect”: high-surface-area nanomaterials can adsorb optical assay reagents, causing false-negative results in standard OECD genotoxicity testing batteries (e.g., micronucleus and comet assays). This mandates the use of appropriate positive controls and orthogonal assay combinations when evaluating the long-term safety of Ind-nanocarriers (123, 188, 189).

A critical observation must be made regarding the methodological quality of the genotoxicity evidence base for Ind nanocarrier systems. The majority of published safety assessments rely on single-generation, short-duration (24–72 h) *in vitro* assays using standard human cell lines or simple invertebrate models. The multigenerational *Daphnia magna* data from Maselli et al. (90) stands as the sole peer-reviewed study employing a chronic, multi-generational toxicological endpoint for an Ind nanocarrier system. This represents a profound evidence asymmetry: while antimicrobial efficacy is typically characterized across multiple strains, concentrations, and experimental formats, genotoxicity is evaluated under conditions that cannot detect the transgenerational effects that chronic environmental exposure would produce. Regulatory agencies are increasingly aware of this asymmetry, and future IND applications for Ind nanocarrier systems should anticipate requirements for extended-duration, multi-generational safety data that the current literature is wholly unprepared to provide.

From a regulatory and environmental perspective, the “safe-by-design” (SbD) concept is emerging as a guiding framework for next-generation AMP-nanocarriers. For Ind systems, SbD translates to specific directives: (i) replacing heavy-metal QD cores with cadmium-free alternatives (124); (ii) designing carrier matrices with defined biodegradation profiles ensuring complete metabolic elimination; and (iii) prioritizing surface chemistries that generate non-toxic degradation products. Furthermore, the One Health framework demands parallel genotoxicity and ecotoxicological evaluations: a formulation demonstrating mammalian safety but exhibiting toxicity in aquatic sentinel organisms or disrupting soil microbiomes is not clinically viable under current environmental regulatory scrutiny (82, 204). In this context, biodegradable organic platforms emerge as superior delivery systems not only pharmacologically, but also as the most responsible choices from a regulatory and environmental perspective. Furthermore, the immunogenicity of Ind as a systemically administered biopharmaceutical has received almost no attention in literature. The potential generation of anti-drug antibodies (ADAs) against Ind following repeated intravenous administration has not been evaluated in any published preclinical study. Given that Ind contains multiple non-natural interactions (D-amino acid analogs in some formulations) and is administered in contexts where immune activation is an intended or likely outcome, ADA formation represents a regulatory safety gap that must be explicitly addressed in future IND applications.

## 8 Comparative analysis of inorganic and organic carrier platforms: a design framework

The available evidence reveals that Ind-nanocarrier systems lie on a continuum between high antimicrobial and immunomodulatory potency, more typical of inorganic platforms, and improved systemic biocompatibility, which is characteristic of organic carriers. Inorganic systems such as AuNPs, AgNPs, CNTs, and QDs exploit their high surface area and intrinsic antimicrobial activity to increase local peptide concentration and facilitate membrane disruption, intracellular delivery, and biofilm penetration. However, they are also more prone to generate ROS, induce DNA damage, or accumulate in organs if size and surface chemistry are not carefully tuned (72–74, 82). Lipid-coated inorganic NPs can retain enhanced antimicrobial properties while the organic shell mitigates protein corona formation, a critical advantage given that, within seconds of exposure to biological fluids, a complex multilayer of plasma proteins adsorbs onto bare nanoparticle surfaces, dramatically altering pharmacokinetic behavior. This hybrid design thus bridges the inorganic-organic dichotomy by addressing the corona problem at the system level while preserving potency locally (115, 116, 178).

In contrast, organic carriers prioritize biocompatibility and controlled release. Liposomes and other lipid-based nanoparticles (including cubosomes) encapsulate cationic peptides within or on the surface of bilayer structures, shielding them from serum proteins and proteases, minimizing nonspecific interactions with host membranes, and enabling sustained local release at the site of infection (48, 55, 56, 205). The selection of an optimal carrier platform must account for multiple clinically relevant variables. The infection type (planktonic vs. biofilm; localized vs. systemic; Gram-positive vs. Gram-negative vs. fungal) fundamentally dictates whether membrane-disrupting potency or intracellular penetration is prioritized (72, 125, 126). Similarly, the administration route (topical, intravenous, inhalational, or mucosal) imposes strict physicochemical constraints on particle size, charge, and enzymatic stability (127–129). The acceptable toxicity threshold is furthermore highly patient-dependent: immunocompromised, pediatric, or critically ill populations require substantially tighter safety margins than immunocompetent adults (129–131). Finally, regulatory feasibility and large-scale manufacturing reproducibility are critical prerequisites for clinical translation that are differentially achievable across inorganic and organic platforms.

Synthesizing these considerations, inorganic conjugates are ideal for topical or localized applications requiring maximum potency and biofilm eradication (74–76), whereas organic systems are superior for systemic delivery where toxicity mitigation and pharmacokinetic protection are the primary concerns (48, 132). The choice of platform must therefore be strictly aligned with the clinical context and therapeutic objectives, not selected on the basis of antimicrobial potency alone.

### 8.1 Evidence quality and the Ind-specificity problem

The comparative framework presented in this section must be interpreted with an important caveat: the quality and Ind-specificity of the evidence underpinning each platform varies considerably. Of the platform-Ind combinations discussed in this review, only three have achieved *in vivo* validation using Ind or a close analog as the active payload: (1) liposomal Ind in a murine systemic aspergillosis model (57); (2) GO-Ind in a murine disseminated candidiasis model (87); and (3) microrobotic Ind delivery in a murine skin infection model (93). All remaining platform-Ind combinations, AgNPs, AuNPs, QDs, CNTs, cubosomes, polymeric nanoparticles, hydrogels, have been validated exclusively through *in vitro* experiments, in model organisms (*Daphnia magna, Galleria mellonella*), or through mechanistic inference from non-Ind AMP systems. Table 2 systematically maps the current state of evidence for each platform-Ind combination across five critical translational dimensions: *in vitro* antimicrobial validation, Ind-specificity of data, *in vivo* efficacy validation, *in vivo* safety validation, and pharmacokinetic characterization.

**Table 2:** Summary of key studies on indolicidin-loaded nanocarrier systems.

Nanocarrier type	Composition	Physicochemical characteristics	Loading strategy	Biological model	Main findings	Advantages/Limitations	References
Gold nanoparticles (AuNPs)	Indolicidin-Cys conjugate+AuNPs (thiol chemistry)	Size: ∼143 nm; Zeta potential (ZP): −4.79 to −4.70 mV	Covalent thiol-gold bonding (cysteine-terminated Ind)	*C. albicans* (fluconazole-resistant clinical isolates), human PBMCs	99.9% viability reduction; downregulation of *ERG11*	Advantages: Bypasses azole resistance; enhanced photostability. Limitations: Requires cysteine engineering; potential immunogenicity.	(41)
AuNPs	Indolicidin+AuNPs (EDC/NHS chemistry)	Size not specified; spherical morphology	EDC/NHS covalent coupling	THP-1 macrophages, *S. typhimurium*	Induces regulatory/anti-inflammatory response.	Advantages: Potent immunomodulatory. Limitations: Complex conjugation chemistry; batch variability.	(75)
AuNPs	Indolicidin+AuNPs (thiol chemistry)	Size: 5–15 nm ZP: ∼-15 mV	Thiol-mediated covalent attachment	*C. albicans* biofilms (clinical isolates). *S. cerevisiae* (genotoxicity model)	4–8-fold improvement against fluconazole-resistant biofilms. Reduced DNA damage compared to bare cationic AuNPs	Advantages: Biofilm penetration via EPS diffusion; Partial biosafety improvement. Limitations: Surface density; Genotoxicity is not completely eliminated and requires optimization.	(45, 68)
Silver nanoparticles (AgNPs)	Indolicidin+carboxylated AgNPs	Size, conjugate: ∼50 nm (includes a 12–15 nm coating)	Physical interactions+encapsulation/ gelatin matrix	*E. coli, Klebsiella spp., P. aeruginosa*, and *S. aureus*	4-fold increased activity against *E. coli, P. aeruginosa*, and *S. aureus*	Advantages: Species-selective potentiation. Limitations: Variable efficacy; Ag^+^ toxicity	(74)
AgNPs	Indolicidin+AgNPs (colloidal, hydrazine reduction)	Size: ∼5.9 nm;	Hydrazine reduction (*in situ* conjugation)	*E. coli, P. aeruginosa* (oral pathology)	Potent activity against oral pathogens	Advantages: Simple synthesis; dental applications. Limitations: Poorly characterized	(78)
AgNPs	Indolicidin+AgNPs (pristine vs. coated)	Size: ∼15 nm	Hydrazine reduction (*in situ* conjugation)	*D. magna* (aquatic ecotoxicity), plant models	Coated AgNPs showed improved ecotoxicological profile; reduced *D. magna* mortality	Advantages: Surface passivation improves safety. Limitations: Long-term environmental fate unknown	(82)
Quantum dots (QDs)	Indolicidin+CdSe/ZnS QDs (amine-functionalized)	Size:∼175.50 nm. Stable across pH 2–10	EDC/NHS covalent linkage	*E. coli, S. aureus*, *D. magna* (acute)	Improved antibacterial activity vs. bare QDs; reduced acute mortality	Advantages: Theranostic potential. Limitations: Heavy metal (Cd^2+^) toxicity; multigenerational effects	(72)
QDs	Indolicidin+CdSe/ZnS QDs	Size:∼175.50 nm. Stable across pH 2–10	EDC/NHS conjugation	*D. magna* (multigenerational, 3 generations)	Multigenerational reproductive decline; DNA alterations; stress gene upregulation; *Vtg* downregulation	Advantages: None for long-term. Limitations: Severe chronic genotoxicity; reproductive toxicity	(90)
Carbon nanotubes (CNTs)	Indolicidin+carboxylated CNTs	Size: outer diameter 8 nm, inner diameter 2–5 nm, length ∼500–2000nm	EDC/NHS covalent coupling	THP-1 macrophages, *S. typhimurium*	Activation of TNFRSF1A/NFkB/c-JUN pathway; protection at subtherapeutic concentrations	Advantages: High aspect ratio for cell penetration; π-π stacking with Trp residues. Limitations: Pulmonary toxicity; poor biodegradability	(76)
CNTs	Indolicidin+carboxylated CNTs	Size: outer diameter 8 nm, inner diameter 2–5 nm, length ∼500–2000nm	EDC/NHS covalent coupling	RAW 264.7, THP-1 macrophages, *S. typhimurium*	CNT-Ind: 1,000-fold lower dose (0.02 µg/mL) enhances immunostimulation & *Salmonella* protection vs. free Ind	Advantages: Immunomodulatory potentiation. Limitations: Pulmonary toxicity; poor biodegradability	(75)
Graphene oxide (GO)	Indolicidin+GO nanocomposite	Honeycomb-structured sheets with hydroxyl and epoxy groups	EDC/NHS covalent coupling	*In vivo* BALB/c murine model of disseminated candidiasis	Potent *in vivo* antifungal activity, minimized toxicity, and pH-responsive release	Advantages: Long-term structural stability; enhanced cellular uptake of Ind. Limitations: Bioaccumulation in the liver and spleen	(87)
Metal oxide NPs	Indolicidin+TiO_2_ or ZnO NPs	3D stable metal oxide crystals	Colloidal suspension mediated by weak electrostatic forces	*P. aeruginosa, K. pneumoniae, A. baumannii* (drug-resistant)	Synergistic mechanism: Photocatalytic ROS ge combined+Ind membrane disruption	Advantages: Broad-spectrum synergy without chemical synthesis overhead Limitations: Poor antimicrobial efficacy of isolated TiO_2_ and ZnO nanoparticles	(88)
Mesoporous silica NPs (MSNPs)	Indolicidin+MSNPs (bare or organically modified)	Size: ∼127.4 nm; ZP: ∼-33.4 mV	Physical mixing/Co-administration with drug-loaded MSNs	MRSA planktonic cultures (*in vitro*)	Synergistic antibacterial activity against MRSA; minimized toxicity (HEK-293 cells) due to dose reduction	Advantages: GRAS status; Synergistic antibacterial activity against MRSA. Limitations: Limited spectrum of synergy	(100)
Liposomes	Indolicidin+POPC liposomes	Size: ∼98–118 nm; neutral	Physical encapsulation (passive loading)	*In vitro:* CHO/K1 cells and human erythrocytes; Balb/c mouse (systemic aspergillosis)	16-fold increase in IC_50_; Maximum tolerated dose *in vivo* elevated 100-fold (from 0.4 mg/kg to 40 mg/kg with 0% mortality).	Advantages: Expanded the therapeutic index Limitations: Premature leakage during storage; lack of peptide protection against proteolytic degradation	(57)
Liposomes	Indolicidin+PEGylated DSPC liposomes	Size: ∼121 nm; ZP: ∼-3.05 mV	Encapsulation- Modified thin-film hydration	*In vitro* HaCaT cell cultures infected with HSV-1	Liposomal encapsulation failed to reduce Ind's cytotoxicity	Limitations: Encapsulation offered no protection against Ind's non-specific toxicity	(48)
Cubosomes	Indolicidin+monoolein (MO)	Size: ∼110–300 nm; ZP: ∼36.2 mV Bicontinuous cubic phase-water channel network	Encapsulation by codissolution	*In vitro* antibacterial assays (*E. coli* and MRSA)	Cubosome encapsulation of Ind did not improve antimicrobial efficacy	Advantages: High encapsulation efficiency. Limitations: MO-based cubic hinder the Ind's ability to inhibit bacterial growth	(104)
Cubic phase lipid	Indolicidin+Phytantriol (PT)	Size: ∼270–345 nm; ZP: Not determined	Solvent evaporation and mechanical dispersion	*In vitro* antibacterial assays (*S. aureus, B. cereus, E. coli*, *P. aeruginosa*)	PT cubosomes maintained/enhanced activity	Advantages: PT-based formulations synergistically enhance activity against bacteria. Limitations: MO cubic nanoparticles hinder the Ind's ability to inhibit bacterial growth.	(86)
Carboxymethyl chitosan (CMCS) + poly(vinyl alcohol) (PVA)	Indolicidin+CMCS+PVA (microspheres, MS)	Size: ∼959 nm ZP:∼-15.7 to −19.8 mV	NHS-amidation	*S. aureus* and *E. coli*; human lung fibroblasts (WI-38); macrophages (RAW 264.7)	Ind-MSs show enhanced antibacterial activity with low toxic effect on lung cells	Advantages: Dual peptide functionalization (lung cell targeting+antimicrobial). Limitations: Low drug loading capacity.	(110)
CMCS+PVA	Indolicidin+CMCS+PVA (microcapsule, CS)	Size: ∼965 nm ZP: Not determined	EDAC or DMT-MM/NHS	WI-38; RAW 264.7	Ind-CS show low toxic effect on lung cells; stimulation of macrophage proliferation	Advantages: Hollow capsules with a higher drug load; Functionalized with two peptides. Limitations: Relatively wide polydispersity	(109)
Hydrogels. PEG-dithiol/ HANor	Indolicidin+PEG hydrogel	HANor/peptide thiol-norbornene click	Crosslinked HANor matrix; swellable network	*In vitro* MRSA adhesion and biofilm formation model	The binding of Ind to its MIC (43.8 µM) reduces bacterial viability and prevents biofilm formation.	Advantages: Localized topical delivery. Limitations: Requires direct contact; not systemic	(111)
Microrobots (PM/Pt-based)	Indolicidin-functionalized microrobots	Size free: 30 µm; ZP: −33.4 mV self-propelled	Covalent Surface functionalizat+Ind via tosyl displacement+Pt sputtering	MRSA biofilms (mature)	Autonomous navigation into biofilm; mechanical disruption and penetration of the EPS; inhibition of topoisomerase I.	Advantages: Active transport; combines mechanical+chemical killing. Limitations: Early-stage; scale-up challenges	(92)
Omiganan-hydrazone-Dex@HA nanoparticles	Omiganan (Ind analog) + Dex, self-assembled PLGA,+HA coating	Size: ∼96.6 nm; ZP: −32.7 mV; loading 62.5%omiganan/13.89%Dex	Acid-labile hydrazone bonds + PLGA-assisted self-assembly + HA targeting	Murine sepsis model and sepsis induced by *S. aureus* and *K. pneumoniae*	Life-saving efficacy in sepsis; synchronized antimicrobial+anti-inflammatory action	Advantages: Responds to infection microenvironment (pH + hyaluronidase+ROS); Co-delivery. Limitations: Complex multi-component system	(132)

Dex, Dexamethasone; DSPC, 1,2-distearoyl-sn-glycero-3-phosphocholine; EDC, 1-ethyl-3-(3-dimethylaminopropyl)carbodiimide; EPS, extracellular polymeric substance; GRAS, Generally Recognized as Safe; LCST, lower critical solution temperature; MIC, minimum inhibitory concentration; MRSA, methicillin-resistant *Staphylococcus aureus*; HA, hyaluronic acid; HANor, norbornene-modified hyaluronic; NHS, N-hydroxysuccinimide; PBMC, peripheral blood mononuclear cell; PLGA, poly(lactic-co-glycolic acid); PM/Pt-based, magnetic particles coated with platinum; POPC, 1-palmitoyl-2-oleoyl-glycero-3-phosphocholine; ROS, reactive oxygen species; SL, Stearylamine Liposomes; SPR, surface plasmon resonance.

This evidence hierarchy must inform clinical translation timelines: liposomal Ind has the strongest translational foundation and should be prioritized for GMP formulation development; GO-Ind and microrobotic Ind represent compelling proof-of-concept systems requiring systematic *in vivo* pharmacokinetic and safety characterization; and all remaining platforms require Ind-specific *in vivo* validation as a prerequisite for any translational claim.

## 9 Synergistic antimicrobial mechanisms and evasion of bacterial resistance

The conjugation of AMPs with nanoparticles produces synergistic antimicrobial effects that significantly amplify efficacy against MDR pathogens (133, 134). This combinatorial approach enables the nanosystem to target multiple biological pathways simultaneously, critically overcoming the “bottleneck” of single-target antibiotic resistance. Nanomaterials contribute intrinsic antimicrobial actions, including severe oxidative stress through ROS generation and physical disruption of bacterial membranes, that complement the peptide's activity (135, 136, 191). The quantification of synergy between AMP-nanocarrier systems and conventional antibiotics is typically performed using the Chou-Talalay combination index (CI) method or checkerboard assays to calculate the Fractional Inhibitory Concentration Index (FICI); a FICI < 0.5 is considered indicative of strong synergy (137, 138). These quantitative frameworks constitute a prerequisite for rigorous preclinical validation and rational combination therapy design.

A fundamental limitation of the existing synergy literature for Ind-nanocarrier systems the near-universal use of planktonic, exponential-phase bacterial cultures in standardized broth microdilution assays. While this provides reproducible and comparable MIC values, it does not capture the behavior of clinically relevant bacterial phenotypes: stationary-phase tolerators, biofilm-embedded cells, intracellular pathogens, and persister cells. For Ind specifically, whose dual mechanism involves both membrane disruption and intracellular topoisomerase I inhibition, the relative contribution of each mechanism, and therefore the synergistic potential of each nanocarrier type, may differ dramatically between planktonic and biofilm contexts. No published study has systematically evaluated the mechanism-specificity of Ind-nanocarrier synergy across these different bacterial physiological states. Until such data exist, FICI values reported in the literature for Ind-nanocarrier systems should be interpreted as planktonic-culture approximations rather than clinically predictive synergy metrics.

This synergy is particularly effective in bypassing complex bacterial defense mechanisms, including enzymatic inactivation and efflux pump overactivation. With respect to efflux pumps, one of the most clinically significant resistance mechanisms in Gram-negative pathogens such as *P. aeruginosa* (MexAB-OprM, MexCD-OprJ) and *E. coli* (AcrAB-TolC), cationic Ind and Ind-loaded nanoparticles can indirectly inhibit pump activity by dissipating the proton motive force (PMF) that energizes these transporters (139, 140). Since the PMF is derived from the membrane potential that Ind's membrane-disrupting activity collapses, Ind-NP systems simultaneously deliver the active peptide to the intracellular target and dismantle the primary efflux resistance mechanism, creating a double vulnerability that dramatically reduces the probability of high-level resistance emergence (17, 141).

It should be noted, however, that this PMF-collapse mechanism for indirect efflux pump inhibition by Ind has not been directly demonstrated for Ind-nanocarrier conjugates using pump activity assays (e.g., NPN fluorescence, ethidium bromide accumulation, or carbonyl cyanide m-chlorophenylhydrazone controls). Existing evidence is largely indirect, inferred from MIC reductions against pump-overexpressing strains without mechanistic confirmation. Direct demonstration of Ind-mediated PMF collapse using electrochemical membrane potential measurements in the context of nanoconjugate delivery represents a meaningful and technically achievable experimental validation priority.

By delivering active peptides directly into the intracellular space or protecting them from enzymatic hydrolysis, nanosystems restore the potency of peptides that would otherwise be inactivated (134). Furthermore, these platforms penetrate the dense extracellular polymeric matrix of bacterial biofilms, disrupting quorum sensing and eradicating persistent colonies within the biofilm structure. AMP-loaded nanoparticles thereby drastically reduce the likelihood of bacteria developing structural resistance, enabling high therapeutic impact at lower systemic doses (133, 136).

## 10 Translational challenges and optimization of AMP-nanocarriers

Despite the immense therapeutic promise of AMP-nanocarrier systems, their transition from laboratory development to clinical application is hindered by formidable pharmacological and toxicological challenges (142, 143). A primary hurdle is ensuring the long-term structural stability of AMPs on nanocarrier surfaces in complex physiological fluids. Unprotected, encapsulated or conjugated peptides are susceptible to premature desorption, proteolytic degradation, and rapid systemic clearance before reaching the target site (144).

For cationic Ind-nanocarrier conjugates, the adsorption of opsonins (immunoglobulins, complement proteins) triggers rapid mononuclear phagocyte system (MPS) uptake by liver and spleen Kupffer cells, reducing circulating half-life and diverting the therapeutic dose from the target infection site (177). Strategies to mitigate corona formation include dense PEGylation, zwitterionic surface coatings (e.g., phosphorylcholine, carboxybetaine), and biomimetic membrane camouflage using erythrocyte or macrophage membrane vesicles (145–147).

Equally critical is the risk of inherent cytotoxicity and off-target effects associated with these hybrid platforms. Since most AMPs rely on their highly cationic nature to disrupt bacterial envelopes, they can inadvertently interact with the negatively charged components of mammalian cell membranes, leading to hemolysis and host tissue damage (65). Nanomaterials, whether polymeric, lipid-based, or inorganic, can accumulate in vital organs if size and surface charge are not rigorously optimized, potentially triggering adverse immunological responses or long-term cellular toxicity (148). Physiologically-based pharmacokinetic (PBPK) modeling has emerged as an important computational tool for predicting NP biodistribution and organ-specific accumulation, enabling rational pre-clinical study design and bridging the translational gap between animal models and human pharmacokinetics (149, 206).

Current FDA and EMA guidelines for nanomedicine require comprehensive characterization of physicochemical properties, including hydrodynamic diameter, polydispersity index (PDI < 0.2), zeta potential, surface chemistry, drug loading efficiency, and *in vitro* release kinetics, as well as a full battery of *in vitro* and *in vivo* safety studies before Investigational New Drug (IND) application submission (121, 122, 150). Beyond biocompatibility, achieving a consistent, sustained, and stimuli-responsive release profile is technically challenging. The local physiological microenvironment at infection sites, characterized by drastic fluctuations in pH, temperature, and enzymatic activity, heavily influences drug delivery kinetics and can compromise the bioavailability and biofilm-penetrating capacity of nanosystems (151, 152). The immunogenicity of the AMP component also requires careful evaluation: repeated systemic administration may trigger anti-drug antibody (ADA) responses that neutralize the therapeutic agent and cause severe hypersensitivity reactions. For IND specifically, the generation of IND-specific IgE antibodies following repeated exposure has not been systematically evaluated in clinical settings, representing an important safety gap that must be addressed through immunogenicity studies in non-human primates prior to Phase I clinical trial initiation.

An overarching translational challenge that warrants explicit acknowledgment is the absence of any published GMP-grade manufacturing protocol for any Ind nanocarrier formulation. GMP manufacture of lipid nanoparticles requires validated, scalable production processes with defined critical quality attributes (CQAs) and in-process controls. For Ind-specific systems, the high membrane affinity of the peptide creates manufacturing-specific risks: adsorption of Ind to process equipment surfaces (tubing, mixers, filters), loss of peptide during sterile filtration, and concentration-dependent aggregation during lyophilization have not been characterized for any Ind nanocarrier platform. These manufacturing uncertainties represent a translational gap that is upstream of the pharmacokinetic and safety gaps discussed in the original section, and they must be addressed in any serious pre-IND program for Ind nanocarrier systems.

## 11 Advanced stimuli-responsive and intelligent nanosystems for controlled Ind delivery

The most advanced frontier in Ind delivery utilizes intelligent systems engineered to release therapeutic payloads strictly in response to precise pathological triggers, such as acidic pH, elevated metalloproteinases, or hyaluronidases, at infection sites. To contextualize these advances clinically, it is instructive to examine the translational trajectory of the payload itself. Omiganan (MBI-226), a 12-residue synthetic Ind derivative, represents the most clinically advanced Ind analog to date. Having progressed through Phase III clinical trials for the prevention of catheter-related bloodstream infections (without achieving regulatory approval for this indication) and Phase II trials for rosacea, it has established critical safety and pharmacokinetic benchmarks for Ind-based therapeutics (5, 6, 153). Historically, despite its optimized structural profile, the systemic administration of omiganan has been heavily precluded by rapid plasma degradation and dose-limiting toxicity (5, 6, 153). The systemic administration of omiganan has been precluded by rapid plasma degradation and dose-limiting toxicity, the same pharmacological barriers that intelligent nanotechnology is designed to overcome by enabling spatiotemporally controlled release of Ind analogs at the site of infection, thereby circumventing deleterious systemic exposure (183).

He et al. (132) exemplified this responsive design by developing a highly sophisticated dual pH- and enzyme-responsive nanosystem in which omiganan was conjugated to dexamethasone via acid-labile hydrazone bonds and coated with hyaluronic acid to target inflamed endothelium. By responding exclusively to the acidic microenvironment and elevated hyaluronidase levels characteristic of severe infection, this formulation ensures the peptide is released in its active, free state precisely where needed (132). The simultaneous co-delivery of a potent immunomodulator (dexamethasone) alongside a bactericidal AMP addresses a critical unmet clinical need in severe systemic infections: while the omiganan payload eradicates the bacterial pathogen, the synchronized, localized release of the corticosteroid actively suppresses the deleterious host hyperinflammatory cascade responsible for the widespread tissue damage characteristic of septic shock. This formulation, displaying life-saving efficacy in murine sepsis models, illustrates how tailored organic surface engineering can transition Ind nanoformulations from passive delivery vehicles to sophisticated, multifunctional therapeutic platforms (132, 154).

The omiganan clinical experience carries an important lesson for intelligent nanocarrier design: even a structurally optimized Ind analog failed in systemic administration due to degradation and toxicity, the same pharmacological barriers that nanocarrier systems are designed to overcome. This clinical precedent argues strongly that the most urgent application of stimuli-responsive Ind nanocarriers is systemic delivery for severe infections (sepsis, invasive fungal disease), where the unmet medical need is greatest and where the toxicity-management advantage of controlled release is most consequential. Topical applications, while technically simpler and closer to clinical readiness, address a less acute unmet need, as several approved topical antimicrobial agents already exist. Future stimuli-responsive Ind nanocarrier development should therefore be explicitly prioritized toward systemic infection indications, even though this pathway entails substantially greater regulatory complexity.

Pushing spatiotemporal control further, the integration of photosensitizers (e.g., rose bengal, porphyrins) into Ind-loaded nanoparticles provides an externally triggered optical switch. These light-activated dual-action platforms simultaneously produce cytotoxic singlet oxygen (photodynamic therapy, PDT) and release Ind upon photoexcitation, creating a highly controlled antimicrobial burst that is exceptionally valuable for eradicating resilient biofilms at wound surfaces or endoscopy-accessible mucosal sites without risking systemic exposure (155, 156).

Finally, the rational design of the Ind payload itself is being revolutionized by artificial intelligence (AI) and machine learning (ML). Deep learning models trained on comprehensive AMP databases, such as APD3, DBAASP, and CAMPR4, can now accurately predict the antimicrobial potency, hemolytic toxicity, and protease resistance of novel Ind sequence variants *in silico* (192). This approach enables the rapid screening of millions of peptide sequences and dramatically compresses the design–synthesize–test cycle (157–160). This computational approach identifies structurally optimized analogs perfectly suited for integration into next-generation intelligent nanocarriers, marking a paradigm shift from empirical to rational AMP engineering (157–161).

## 12 Perspectives and conclusions

### 12.1 Critical assessment of the field's current state

Although the strategic integration of nanotechnology with indolicidin demonstrates profound synergistic effects, particularly in bypassing efflux-mediated resistance mechanisms and penetrating dense biofilms, translating these experimental systems into clinical settings presents formidable translational barriers. Long-term structural stability, prevention of premature peptide desorption in complex physiological fluids, and biopersistence of nanomaterials remain major regulatory concerns.

**Figure 4:**
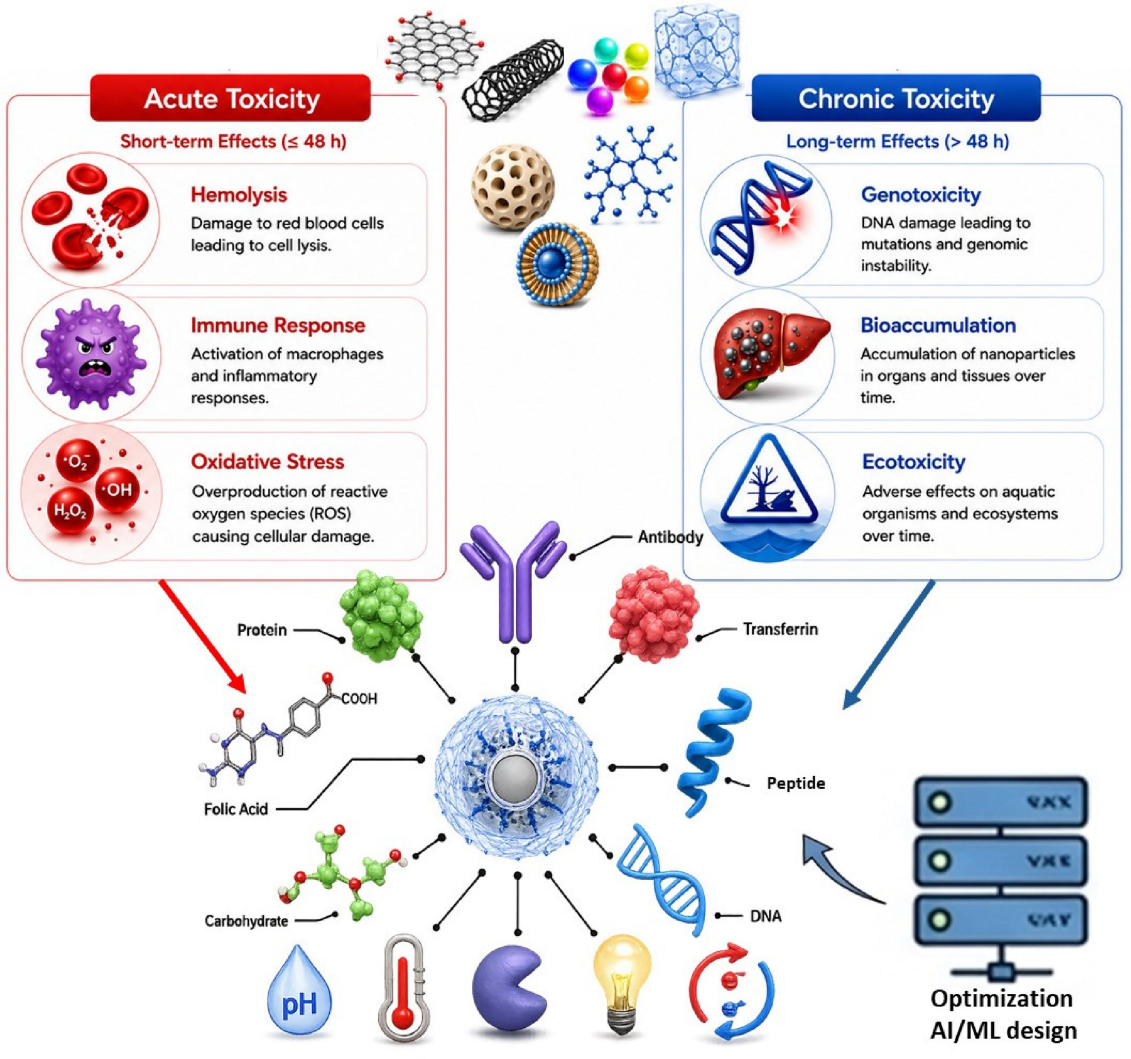
Integrated toxicological and design framework for indolicidin (Ind) nanocarrier systems: from acute physiological responses to chronic bioaccumulation barriers and rational optimization strategies. Comparative toxicological overview of Ind nanoconjugates across different biological fates and safety profiles of organic (left) and inorganic (right) platforms over varying time periods. Acute toxicity (0-48 h): Physiological responses to organic platforms are characterized by transient membrane interactions, potential hemolytic bursts, and rapid opsonin-mediated elimination. Inorganic platforms can mitigate initial peptide-related hemolysis but trigger oxidative stress early on. Chronic toxicity (weeks to months): Illustrates long-term pharmacological challenges where inorganic cores exhibit a toxicological delay, facilitating prolonged bioaccumulation in the reticuloendothelial system.Safety implications: The diagram underscores the need for optimization of “smart” nanoparticle systems using bioinformatics and “AI” platforms. (Figure created with AI).

As highlighted throughout this analysis, Ind-conjugated nanosystems exhibit a “toxicological paradox”: while nanoformulation successfully mitigates acute toxicity and immediate hemolysis, multigenerational models reveal hidden risks of genotoxicity and transgenerational reproductive decline. This discrepancy exposes a critical gap in current safety protocols, demonstrating that standard 24–48-hour *in vitro* cytotoxicity assays are insufficient to predict the long-term biological impact of nanocarriers. As illustrated in Figure 4, the toxicological landscape of Ind-nanosystems is fundamentally bifurcated by the physicochemical nature of the carrier. Organic platforms, predominantly liposomes, cubosomes, and biodegradable polymers, exhibit a profile dominated by acute physiological interactions, such as transient hemolysis and rapid opsonin-driven clearance, which typically resolve through established metabolic pathways; conversely, inorganic platforms (e.g., quantum dots, carbon nanotubes, and metallic nanoparticles) present unique risks of persistent intracellular accumulation, chronic oxidative stress, and latent genotoxicity.

It is therefore imperative to adopt comprehensive chronic and ecotoxicological evaluations aligned with OECD Test Guidelines (e.g., TG 487 for *in vitro* micronucleus tests and TG 476 for gene mutation tests) and ISO nanomaterial safety standards. The clinical success of Ind-based nanomedicines should not be measured solely by bactericidal potency, but by the guarantee that their biopersistence does not compromise translational pipelines, which must integrate chronic environmental and genetic assessments.

To address these limitations and align with a One Health perspective, future research must prioritize highly biocompatible, stimuli-responsive matrices, such as enzyme-cleavable hydrogels or hybrid lipid-polymer architectures, and supramolecular nanostructures that ensure both the controlled localized release of Ind and the safe metabolic elimination of the carrier (204). The confluence of AI-guided peptide sequence optimization (predicting and enhancing protease resistance), environmentally benign nanotechnology, and precision stimuli-responsive delivery systems positions Ind-nanocarrier platforms at the vanguard of next-generation antimicrobial therapeutics.

### 12.2 Priority research agenda for Ind nanocarrier development

Based on the analysis presented herein, the following research priorities are identified, listed in approximate order of urgency for translational advancement:

1. Systematic *in vivo* pharmacokinetic characterization: Without plasma t_1/2_, AUC, C_max_, and tissue distribution data for Ind as the encapsulated or conjugated payload, rather than as a model drug, no meaningful translational claim can be made for any platform. This gap represents the most critical roadblock to clinical approval.
2. Head-to-head comparison of conjugation modalities: A study directly comparing encapsulated, non-cleavably conjugated, and stimuli-responsively conjugated Ind against the same MDR pathogen panel, using standardized nanocarrier core material, would empirically validate the mechanistic framework proposed in Section 3 and constitute a landmark contribution to the field.
3. *In vivo* validation of inorganic platforms in mammalian infection models: Of the eight inorganic platforms discussed, only two have undergone *in vivo* mammalian validation for Ind. AgNP-Ind, CNT-Ind, QD-Ind, and MSNP-Ind systems require *in vivo* proof-of-concept evaluations before their translational potential can be accurately assessed.
4. Multi-generational safety studies for promising formulations: As demonstrated by the QD-Ind case, *in vitro* safety assays are insufficient for regulatory characterization. Extended chronic and ecotoxicological evaluation should accompany *in vivo* efficacy validation from the earliest stages of development.
5. GMP development for advanced formulations: The liposomal Ind system developed by Ahmad et al. (57) has the strongest translational evidence base and is the most appropriate candidate for GMP formulation development, scale-up, and formal pharmacokinetic/pharmacodynamic (PK-PD) modeling in preparation for Phase I clinical trial design.
6. AI-guided sequence optimization for nanocarrier compatibility: Deep learning models should be trained not only to optimize Ind's antimicrobial potency and protease resistance, but explicitly to predict compatibility with specific nanocarrier architectures, minimizing cubosomal phase disruption, maximizing controlled-release loading efficiency, and predicting surface density-dependent toxicity profiles.

In summary, this review establishes that: (i) the pharmacodynamic outcome of Ind nanoconjugation is fundamentally determined by the conjugation paradigm (encapsulation vs. covalent vs. cleavable tethering); (ii) inorganic platforms provide superior potency and biofilm penetration but carry significant genotoxicological liabilities that must be addressed through safe-by-design principles; (iii) organic and polymeric systems offer the most favorable translational safety profile and are best suited for systemic applications; and (iv) advanced stimuli-responsive and AI-guided platforms represent the next frontier for overcoming both bacterial resistance and host toxicity simultaneously. Successful clinical translation will ultimately require sustained investment in GMP manufacturing, robust pharmacokinetic–pharmacodynamic (PK-PD) modeling, and comprehensive Phase I-III clinical trials evaluating both efficacy against defined MDR pathogens and the full spectrum of acute, chronic, and environmental safety endpoints. By matching this versatile peptide with intelligently engineered nanocarriers, researchers can fully exploit its therapeutic potential to overcome the global crisis of antimicrobial resistance.

## References

[1] Alzain M Daghistani H Shamrani T Almoghrabi Y Daghistani Y Alharbi OS. Antimicrobial peptides: mechanisms, applications, and therapeutic potential. Infect Drug Resist. (2025) 18:4385–426. 10.2147/IDR.S514825 40901006 PMC12399857

[2] del Olmo ML Andreu C. Current Status of the application of antimicrobial peptides and their conjugated derivatives. Molecules. (2025) 30(15):3070. 10.3390/molecules30153070 40807245 PMC12348590

[3] Subbalakshmi C Krishnakumari V Sitaram N Nagaraj R. Interaction of indolicidin, a 13-residue peptide rich in tryptophan and proline and its analogues with model membranes. J Biosci. (1998) 23(1):9–13. 10.1007/BF02728517

[4] Marchand C Krajewski K Lee HF Antony S Johnson AA Amin R. Covalent binding of the natural antimicrobial peptide indolicidin to DNA abasic sites. Nucleic Acids Res. (2006) 34(18):5157–65. 10.1093/nar/gkl667 16998183 PMC1636436

[5] Melo MN Dugourd D Castanho MA. Omiganan pentahydrochloride in the front line of clinical applications of antimicrobial peptides. Recent Pat Anti-Infect Drug Discovery. (2006) 1(2):201–7. 10.2174/157489106777452638 18221145

[6] Sader HS Fedler KA Rennie RP Stevens S Jones RN. Omiganan pentahydrochloride (MBI 226), a topical 12-amino-acid cationic peptide: spectrum of antimicrobial activity and measurements of bactericidal activity. Antimicrob Agents Chemother. (2004) 48(8):3112–8. 10.1128/AAC.48.8.3112-3118.2004 15273128 PMC478492

[7] Ilieş BD Yildiz I Abbas M. Peptide-conjugated nanoparticle platforms for targeted delivery, imaging, and biosensing applications. ChemBioChem. (2024) 25(10):e202300867. 10.1002/cbic.202300867 38551557

[8] Xhindoli D Pacor S Benincasa M Scocchi M Gennaro R Tossi A. The human cathelicidin LL-37—a pore-forming antibacterial peptide and host-cell modulator. Biochimica et Biophysica Acta (BBA)-Biomembranes. (2016) 1858(3):546–66. 10.1016/j.bbamem.2015.11.003 26556394

[9] Tomasinsig L Zanetti M. The cathelicidins-structure, function and evolution. Curr Protein Pept Sci. (2005) 6(1):23–34. 10.2174/1389203053027520 15638766

[10] Brogden KA Ackermann M McCray PB Jr Tack BF. Antimicrobial peptides in animals and their role in host defences. Int J Antimicrob Agents. (2003) 22(5):465–78. 10.1016/s0924-8579(03)00180-8 14602364

[11] Friedrich CL Rozek A Patrzykat A Hancock RE. Structure and mechanism of action of an indolicidin peptide derivative with improved activity against gram-positive bacteria. J Biol Chem. (2001) 276(26):24015–22. 10.1074/jbc.M009691200 11294848

[12] Chang CY Lin CW Chiang SK Chen PL Huang CY Liu SJ. Enzymatic stability and immunoregulatory efficacy of a synthetic indolicidin analogue with regular enantiomeric sequence. ACS Med Chem Lett. (2013) 4(6):522–6. 10.1021/ml400081f 24900703 PMC4027239

[13] Hsu CH Chen C Jou ML Lee AYL Lin YC Yu Y. Structural and DNA-binding studies on the bovine antimicrobial peptide, indolicidin: evidence for multiple conformations involved in binding to membranes and DNA. Nucleic Acids Res. (2005) 33(13):4053–64. 10.1093/nar/gki725 16034027 PMC1179735

[14] Rozek A Friedrich CL Hancock RE. Structure of the bovine antimicrobial peptide indolicidin bound to dodecylphosphocholine and sodium dodecyl sulfate micelles. Biochemistry. (2000) 39(51):15765–74. 10.1021/bi000714m 11123901

[15] Fitz AR Klimov DK Lockhart C. Binding of antimicrobial peptide indolicidin to DMPC bilayer using replica-exchange molecular dynamics. J Chem Inf Model. (2025) 65(17):9251–60. 10.1021/acs.jcim.5c01153 40839696 PMC12421676

[16] Hsu JC Yip CM. Molecular dynamics simulations of indolicidin association with model lipid bilayers. Biophys J. (2007) 92(12):L100–2. 10.1529/biophysj.107.108050 17416617 PMC1877785

[17] Hollmann A Martinez M Maturana P Semorile LC Maffia PC. Antimicrobial peptides: interaction with model and biological membranes and synergism with chemical antibiotics. Front Chem. (2018) 6:204. 10.3389/fchem.2018.00204 29922648 PMC5996110

[18] Neale C Hsu JC Yip CM Pomès R. Indolicidin binding induces thinning of a lipid bilayer. Biophys J. (2014) 106(8):L29–31. 10.1016/j.bpj.2014.02.031 24739184 PMC4008834

[19] Mohid SA Bhunia A. Combining antimicrobial peptides with nanotechnology: an emerging field in theranostics. Curr Protein Pept Sci. (2020) 21(4):413–28. 10.2174/1389203721666191231111634 31889488

[20] Wimley WC Hristova K. Antimicrobial peptides: successes, challenges and unanswered questions. J Membr Biol. (2011) 239(1–2):27–34. 10.1007/s00232-011-9343-0 21225255 PMC3166253

[21] Rokitskaya TI Kolodkin NI Antonenko YN. Effect of antibacterial peptide indolicidin on the membrane permeability: carrier mechanism versus pore formation. Biophys J. (2010) 98(3):107a. 10.1016/j.bpj.2009.12.595 20851098

[22] Ghosh A Kar RK Jana J Saha A Jana B Krishnamoorthy J. Indolicidin targets duplex DNA: structural and mechanistic insight through a combination of spectroscopy and microscopy. ChemMedChem. (2014) 9(9):2052–8. 10.1002/cmdc.201402215 25044630

[23] Gagandeep KR Balenahalli Narasingappa R Vishnu Vyas G. Unveiling mechanisms of antimicrobial peptide: actions beyond the membranes disruption. Heliyon. (2024) 10(19):e38079. 10.1016/j.heliyon.2024.e38079 39386776 PMC11462253

[24] Mookherjee N Anderson MA Haagsman HP Davidson DJ. Antimicrobial host defence peptides: functions and clinical potential. Nat Rev Drug Discov. (2020) 19(5):311–32. 10.1038/s41573-019-0058-8 32107480

[25] Young-Speirs M Drouin D Cavalcante PA Barkema HW Cobo ER. Host defense cathelicidins in cattle: types, production, bioactive functions and potential therapeutic and diagnostic applications. Int J Antimicrob Agents. (2018) 51(6):813–21. 10.1016/j.ijantimicag.2018.02.006 29476808

[26] Jamal M Ahmad W Andleeb S Jalil F Imran M Nawaz MA. Bacterial biofilm and associated infections. J Chin Med Assoc. (2018) 81(1):7–11. 10.1016/j.jcma.2017.07.012 29042186

[27] Smirnova MP Kolodkin NI Kolobov AA Afonin VG Afonina IV Stefanenko LI. Indolicidin analogs with broad-spectrum antimicrobial activity and low hemolytic activity. Peptides. (2020) 132:170356. 10.1016/j.peptides.2020.170356 32593681

[28] Subbalakshmi C Krishnakumari V Nagaraj R Sitaram N. Requirements for antibacterial and hemolytic activities in the bovine neutrophil derived 13-residue peptide indolicidin. FEBS Lett. (1996) 395(1):48–52. 10.1016/0014-5793(96)00996-9 8849687

[29] Vasilchenko AS Vasilchenko AV Pashkova TM Smirnova MP Kolodkin NI Manukhov IV. Antimicrobial activity of the indolicidin-derived novel synthetic peptide in-58. J Pept Sci. (2017) 23(12):855–63. 10.1002/psc.3049 29193518

[30] Friedrich CL Moyles D Beveridge TJ Hancock REW. Antibacterial action of structurally diverse cationic peptides on gram-positive bacteria. Antimicrob Agents Chemother. (2000) 44:2086–92. 10.1128/AAC.44.8.2086-2092.2000 10898680 PMC90018

[31] Jindal HM Le CF Mohd Yusof MY Velayuthan RD Lee SV Zain SM. Antimicrobial activity of novel synthetic peptides derived from indolicidin and ranalexin against *Streptococcus pneumoniae*. PLoS One. (2015) 10(6):e0128532. 10.1371/journal.pone.0128532 26046345 PMC4457802

[32] Falla TJ Karunaratne DN Hancock RE. Mode of action of the antimicrobial peptide indolicidin. J Biol Chem. (1996) 271(32):19298–303. 10.1074/jbc.271.32.19298 8702613

[33] Nan YH Park KH Park Y Jeon YJ Kim Y Park IS. Investigating the effects of positive charge and hydrophobicity on the cell selectivity, mechanism of action and anti-inflammatory activity of a trp-rich antimicrobial peptide indolicidin. FEMS Microbiol Lett. (2009b) 292(1):134–40. 10.1111/j.1574-6968.2008.01484.x 19191872

[34] Nan YH Bang JK Shin SY. Design of novel indolicidin-derived antimicrobial peptides with enhanced cell specificity and potent anti-inflammatory activity. Peptides. (2009a) 30(5):832–8. 10.1016/j.peptides.2009.01.015 19428758

[35] Kim J Lee J Kang E Lee K Lee K Cheon Y. Insights into an indolicidin-derived low-toxic anti-microbial peptide’s efficacy against bacterial cells while preserving eukaryotic cell viability. Biofactors. (2025) 51(1):e2145. 10.1002/biof.2145 39569798

[36] Dwivedi R Aggarwal P Bhavesh NS Kaur KJ. Design of therapeutically improved analogue of the antimicrobial peptide, indolicidin, using a glycosylation strategy. Amino Acids. (2019) 51(10-12):1443–60. 10.1007/s00726-019-02779-2 31485742

[37] Lone ABN Nielsen JE Thulstrup PW Lund R Hansen PR Jenssen H. Cyclic N-locked indolicidin analogues with antimicrobial activity: effect of ring size and fatty acid acylation. Eur J Med Chem Rep. (2022) 6:100080. 10.1016/j.ejmcr.2022.100080

[38] Mohamed MF Brezden A Mohammad H Chmielewski J Seleem MN. A short D-enantiomeric antimicrobial peptide with potent immunomodulatory and antibiofilm activity against multidrug-resistant Pseudomonas aeruginosa and Acinetobacter baumannii. Sci Rep. (2017) 7(1):6953. 10.1038/s41598-017-07440-0 28761101 PMC5537347

[39] Park KH Nan YH Park Y Kim JI Park IS Hahm KS. Cell specificity, anti-inFlammatory activity, and plausible bactericidal mechanism of designed trp-rich model antimicrobial peptides. Biochim Biophys Acta Biomembr. (2009) 1788(5):1193–203. 10.1016/j.bbamem.2009.02.020 19285481

[40] Ngo VH Luong XH Le VH Phuong HBT Do Hai Y Thang NQ. Indolicidin derivatives as potent dual-action antifungal and antibacterial agents for the treatment of skin infections: a comprehensive study from *in vitro* to *in vivo* evaluation. PLoS One. (2025) 20(9):e0331796. 10.1371/journal.pone.0331796 40911560 PMC12412968

[41] Rahimi H Roudbar mohammadi S Delavari HH Roudbary M. Antifungal effects of indolicidin-conjugated gold nanoparticles against fluconazole-resistant strains of Candida albicans isolated from patients with burn infection. Int J Nanomed. (2019) 14:5323–38. 10.2147/IJN.S207527 PMC6646856 31409990

[42] Thu HN Van HN Phuong HBT Duc TP Van KH Vu KN. The importance role of central proline to the antimicrobial potency and selectivity of indolicidin. Arch Microbiol. (2025) 207(4):97. 10.1007/s00203-025-04299-y 40113630

[43] Benincasa M Scocchi M Pacor S Tossi A Nobili D Basaglia G. Fungicidal activity of five cathelicidin peptides against clinically isolated yeasts. J Antimicrob Chemother. (2006) 58:950–9. 10.1093/jac/dkl382 17023499

[44] Giacometti A Cirioni O Barchiesi F Caselli F Scalise G. *In-vitro* activity of polycationic peptides against cryptosporidium parvum, pneumocystis carinii and yeast clinical isolates. J Antimicrob Chemother. (1999) 44:403–6. 10.1093/jac/44.3.403 10511411

[45] de Alteriis E Maselli V Falanga A Galdiero S Di Lella FM Gesuele R. Efficiency of gold nanoparticles coated with the antimicrobial peptide indolicidin against biofilm formation and development of Candida spp. Clinical isolates. Infect Drug Resist. (2018a) 11:915–25. 10.2147/IDR.S164262 30013374 PMC6037145

[46] Cirioni O Giacometti A Barchiesi F Scalise G. *In-vitro* activity of lytic peptides alone and in combination with macrolides and inhibitors of dihydrofolate reductase against pneumocystis carinii. J Antimicrob Chemother. (1998) 42(4):445–51. 10.1093/jac/42.4.445 9818742

[47] Matanic VCA Castilla V. Antiviral activity of antimicrobial cationic peptides against junin virus and herpes simplex virus. Int J Antimicrob Agents. (2004) 23(4):382–9. 10.1016/j.ijantimicag.2003.07.022 15081088

[48] Ron-Doitch S Sawodny B Kühbacher A David MMN Samanta A Phopase J. Reduced cytotoxicity and enhanced bioactivity of cationic antimicrobial peptides liposomes in cell cultures and 3D epidermis model against HSV. J Control Release. (2016) 229:163–71. 10.1016/j.jconrel.2016.03.025 27012977

[49] Yasin B Pang M Turner JS Cho Y Dinh NN Waring AJ. Evaluation of the inactivation of infectious herpes simplex virus by host-defense peptides. Eur J Clin Microbiol Infect Dis. (2000) 19(3):187–94. 10.1007/s100960050457 10795591

[50] Haines LR Hancock RE Pearson TW. Cationic antimicrobial peptide killing of African trypanosomes and Sodalis glossinidius, a bacterial symbiont of the insect vector of sleeping sickness. Vector-Borne Zoonotic Dis. (2003) 3(4):175–86. 10.1089/153036603322662165 14733670

[51] Arias M Haney EF Hilchie AL Corcoran JA Hyndman ME Hancock RE. Selective anticancer activity of synthetic peptides derived from the host defence peptide tritrpticin. Biochimica et Biophysica Acta (BBA)-Biomembranes. (2020) 1862(8):183228. 10.1016/j.bbamem.2020.183228 32126228

[52] Bacalum M. Antimicrobial peptides show antitumor activity against SH-SY-5Y human neuroblastoma cells. In: Florescu M, editor. Biophysics for Biomedical and Environmental Sciences. Brașov: Transilvania University of Brasov Press (2016). p. 39–48.

[53] Vergis J Malik SS Pathak R Kumar M Ramanjaneya S Kurkure NV. Antimicrobial efficacy of indolicidin against multi-drug resistant enteroaggregative Escherichia coli in a galleria mellonella model. Front Microbiol. (2019) 10:2723. 10.3389/fmicb.2019.02723 31849877 PMC6895141

[54] Falla TJ Hancock RE. Improved activity of a synthetic indolicidin analog. Antimicrob Agents Chemother. (1997) 41:771–5. 10.1128/aac.41.4.771 9087487 PMC163792

[55] Ahmad N Bukhari SNA Hussain MA Ejaz H Munir MU Amjad MW. Nanoparticles incorporated hydrogels for delivery of antimicrobial agents: developments and trends. RSC Adv. (2024) 14(19):13535–64. 10.1039/d4ra00631c 38665493 PMC11043667

[56] Forier K Raemdonck K De Smedt SC Demeester J Coenye T Braeckmans K. Lipid and polymer nanoparticles for drug delivery to bacterial biofilms. J Control Release. (2014) 190:607–23. 10.1016/j.jconrel.2014.03.055 24794896

[57] Ahmad I Perkins WR Lupan DM Selsted ME Janoff AS. Liposomal entrapment of the neutrophil-derived peptide indolicidin endows it with *in vivo* antifungal activity. Biochimica et Biophysica Acta (BBA)-Biomembranes. (1995) 1237(2):109–14. 10.1016/0005-2736(95)00087-j 7632702

[58] Carrer M Nielsen JE Cezar HM Lund R Cascella M Soares TA. Accelerating lipid flip-flop at low concentrations: a general mechanism for membrane binding peptides. J Phys Chem Lett. (2023) 14(31):7014–9. 10.1021/acs.jpclett.3c01284 37523748 PMC10424232

[59] Khandelia H Kaznessis YN. Cation−π interactions stabilize the structure of the antimicrobial peptide indolicidin near membranes: molecular dynamics simulations. J Phys Chem B. (2007) 111(1):242–50. 10.1021/jp064776j 17201448 PMC2440664

[60] Smart M Rajagopal A Liu WK Ha BY. Opposing effects of cationic antimicrobial peptides and divalent cations on bacterial lipopolysaccharides. Physical Review E. (2017) 96(4):042405. 10.1103/PhysRevE.96.042405 29347628

[61] Li J Zheng C Cansiz S Wu C Xu J Cui C. Self-assembly of DNA nanohydrogels with controllable size and stimuli-responsive property for targeted gene regulation therapy. J Am Chem Soc. (2015) 137(4):1412–5. 10.1021/ja512293f 25581100 PMC4449038

[62] Braun K Pochert A Linden M Davoudi M Schmidtchen A Nordstrom R. Membrane interactions of mesoporous silica nanoparticles as carriers of antimicrobial peptides. J Colloid Interface Sci. (2016) 475:161–70. 10.1016/j.jcis.2016.05.002 27174622

[63] Brogden NK Brogden KA. Will new generations of modified antimicrobial peptides improve their potential as pharmaceuticals? Int J Antimicrob Agents. (2011) 38(3):217–25. 10.1016/j.ijantimicag.2011.05.004 21733662 PMC3159164

[64] Talapko J Meštrović T Juzbašić M Tomas M Erić S Horvat Aleksijević L. Antimicrobial peptides—mechanisms of action, antimicrobial effects and clinical applications. Antibiotics. (2022) 11(10):1417. 10.3390/antibiotics11101417 36290075 PMC9598582

[65] Huang Y Guo X Wu Y Chen X Feng L Xie N. Nanotechnology’s frontier in combatting infectious and inFlammatory diseases: prevention and treatment. Signal Transduct Target Ther. (2024) 9(1):34. 10.1038/s41392-024-01745-z 38378653 PMC10879169

[66] Dong X Liang W Meziani MJ Sun Y-P Yang L. Carbon dots as potent antimicrobial agents. Theranostics. (2020) 10:671–86. 10.7150/thno.39863 31903144 PMC6929978

[67] Dad N Elsawy MA Humphreys G Pluen A Lu JR McBain A J. A critical view of antimicrobial peptides: exploring their potential and the barriers to realization. J Appl Microbiol. (2025) 136(5):lxaf087. 10.1093/jambio/lxaf087 40205522

[68] de Alteriis E Falanga A Galdiero S Guida M Maselli V Galdiero E. Genotoxicity of gold nanoparticles functionalized with indolicidin towards Saccharomyces cerevisiae. Journal of Environmental Sciences. (2018b) 66:138–45. 10.1016/j.jes.2017.04.034 29628080

[69] He S Deber CM. Interaction of designed cationic antimicrobial peptides with the outer membrane of gram-negative bacteria. Sci Rep. (2024) 14(1):1894. 10.1038/s41598-024-51716-1 38253659 PMC10803810

[70] Kumar K Sebastiao M Arnold AA Bourgault S Warschawski DE Marcotte I. *In situ* solid-state NMR study of antimicrobial peptide interactions with erythrocyte membranes. Biophys J. (2022) 121(8):1512–24. 10.1016/j.bpj.2022.03.009 35278426 PMC9072582

[71] AlQurashi DM AlQurashi TF Alam RI Shaikh S Tarkistani MAM. Advanced nanoparticles in combating antibiotic resistance: current innovations and future directions. Journal of Nanotheranostics. (2025) 6(2):9. 10.3390/jnt6020009

[72] Galdiero E Siciliano A Maselli V Gesuele R Guida M Fulgione D. An integrated study on antimicrobial activity and ecotoxicity of quantum dots and quantum dots coated with the antimicrobial peptide indolicidin. Int J Nanomedicine. (2016) 11:4199–211. 10.2147/IJN.S107752 27616887 PMC5008656

[73] Malmsten M. Inorganic nanomaterials as delivery systems for proteins, peptides, DNA, and siRNA. Curr Opin Colloid Interface Sci. (2013) 18(5):468–80. 10.1016/j.cocis.2013.06.002

[74] Zharkova MS Golubeva OY Orlov DS Vladimirova EV Dmitriev AV Tossi A. Silver nanoparticles functionalized with antimicrobial polypeptides: benefits and possible pitfalls of a novel anti-infective tool. Front Microbiol. (2021) 12:750556. 10.3389/fmicb.2021.750556 34975782 PMC8719061

[75] Sur A Pradhan B Banerjee A Aich P. Immune activation efficacy of indolicidin is enhanced upon conjugation with carbon nanotubes and gold nanoparticles. PLoS One. (2015) 10(4):e0123905. 10.1371/journal.pone.0123905 25876153 PMC4398554

[76] Pradhan B Guha D Murmu KC Sur A Ray P Das D. Comparative efficacy análisis of anti-microbial peptides, LL-37 and indolicidin upon conjugation with CNT, in human monocytes. J Nanobiotechnol. (2017) 15(1):44. 10.1186/s12951-017-0278-1 PMC5469186 28606090

[77] Aguilar-Garay R Lara-Ortiz LF Campos-López M Gonzalez-Rodriguez DE Gamboa-Lugo MM Mendoza-Pérez JA. A comprehensive review of silver and gold nanoparticles as effective antibacterial agents. Pharmaceuticals. (2024) 17(9):1134. 10.3390/ph17091134 39338299 PMC11434858

[78] Zannella C Shinde S Vitiello M Falanga A Galdiero E Fahmi A. Antibacterial activity of indolicidin-coated silver nanoparticles in oral disease. Appl Sci. (2020) 10(5):1837. 10.3390/app10051837

[79] Wan G Ruan L Yin Y Yang T Ge M Cheng X. Effects of silver nanoparticles in combination with antibiotics on the resistant bacteria *Acinetobacter baumannii*. Int J Nanomed. (2016) 11:3789–800. 10.2147/IJN.S104166 PMC4990392 27574420

[80] Dakal TC Kumar A Majumdar RS Yadav V. Mechanistic basis of antimicrobial actions of silver nanoparticles. Front Microbiol. (2016) 7:1831. 10.3389/fmicb.2016.01831 27899918 PMC5110546

[81] McNeilly O Mediati DG Pittorino MJ Mann R Söderström B Hamidian M. Molecular insights into silver nanoparticle resistance in Acinetobacter baumannii and unique adaptations to ionic silver. NPJ Antimicrobials and Resistance. (2025) 3(1):92. 10.1038/s44259-025-00161-9 41291222 PMC12647597

[82] Falanga A Siciliano A Vitiello M Franci G Del Genio V Galdiero S. Ecotoxicity evaluation of pristine and indolicidin-coated silver nanoparticles in aquatic and terrestrial ecosystem. Int J Nanomed. (2020) 15:8097–108. 10.2147/IJN.S260396 PMC7585781 33116520

[83] Colilla M Vallet-Regí M. Organically modified mesoporous silica nanoparticles against bacterial resistance. Chem Mater. (2023) 35(21):8788–805. 10.1021/acs.chemmater.3c02192 38027542 PMC10653088

[84] Vadukumpully S Gupta J Zhang Y Xu GQ Valiyaveettil S. Functionalization of surfactant wrapped graphene nanosheets with alkylazides for enhanced dispersibility. Nanoscale. (2011) 3(1):303–8. 10.1039/C0NR00547A 21052576

[85] Liu Z Tabakman S Welsher K Dai H. Carbon nanotubes in biology and medicine: *in vitro* and *in vivo* detection, imaging and drug delivery. Nano Res. (2009) 2(2):85–120. 10.1007/s12274-009-9009-8 20174481 PMC2824900

[86] Meikle TG Dharmadana D Hoffmann SV Jones NC Drummond CJ Conn CE. Analysis of the structure, loading and activity of six antimicrobial peptides encapsulated in cubic phase lipid nanoparticles. J Colloid Interface Sci. (2021) 587:90–100. 10.1016/j.jcis.2020.11.124 33360913

[87] Farzanegan A Roudbary M Falahati M Khoobi M Gholibegloo E Farahyar S. Synthesis, characterization and antifungal activity of a novel formulated nanocomposite containing indolicidin and graphene oxide against disseminated candidiasis. Journal de Mycologie Médicale. (2018) 28(4):628–36. 10.1016/j.mycmed.2018.07.009 30126717

[88] Masoumi S Shakibaie MR Gholamrezazadeh M Monirzadeh F. Evaluation synergistic effect of TiO2, ZnO nanoparticles and amphiphilic peptides (mastoparan-B, indolicidin) against drug-resistant Pseudomonas aeruginosa, Klebsiella pneumoniae and Acinetobacter baumannii. Arch Pediatr Infect Dis. (2018) 6(3):0–7. 10.5812/pedinfect.57920

[89] Masoumi H Mirfendereski SM. Modification of physical and thermal characteristics of stearic acid as a phase change materials using TiO2-nanoparticles. Thermochim Acta. (2019) 675:9–17. 10.1016/j.tca.2019.02.015

[90] Maselli V Siciliano A Giorgio A Falanga A Galdiero S Guida M. Multigenerational effects and DNA alterations of QDs-indolicidin on daphnia magna. Environ Pollut. (2017) 224:597–605. 10.1016/j.envpol.2017.02.043 28242252

[91] Noga M Milan J Frydrych A Jurowski K. Toxicological aspects, safety assessment, and green toxicology of silver nanoparticles (AgNPs)—critical review: state of the art. Int J Mol Sci. (2023) 24(6):5133. 10.3390/ijms24065133 36982206 PMC10049346

[92] Milosavljevic V Kosaristanova L Dolezelikova K Adam V Pumera M. Microrobots with antimicrobial peptide nanoarchitectonics for the eradication of antibiotic-resistant biofilms. Adv Funct Mater. (2022) 32(43):2112935. 10.1002/adfm.202112935

[93] Jancik-Prochazkova A Michalkova H Cihalova K Heger Z Pumera M. Microrobots for antibiotic-resistant Staphylococcus aureus skin colony eradication. ACS Appl Mater Interfaces. (2025) 17(27):39340–8. 10.1021/acsami.5c08683 40560979 PMC12257449

[94] Jyoti AS Peng X Pumera M. Virus enhanced microrobots for biofilm eradication. Adv Mater. (2026) 38(2):e08299. 10.1002/adma.202508299 41045105 PMC12783983

[95] Memar MY Ghotaslou R Samiei M Adibkia K. Antimicrobial use of reactive oxygen therapy: current insights. Infect Drug Resist. (2018) 11:567–76. 10.2147/IDR.S142397 29731645 PMC5926076

[96] Oyinloye B Adenowo A Kappo A. Reactive oxygen species, apoptosis, antimicrobial peptides and human inflammatory diseases. Pharmaceuticals. (2015) 8(2):151–75. 10.3390/ph8020151 25850012 PMC4491653

[97] de Oliveira KBS Leite ML Melo NTM Lima LF Barbosa TCQ Carmo NL. Antimicrobial peptide delivery systems as promising tools against resistant bacterial infections. Antibiotics (Basel). (2024) 13(11):1042. 10.3390/antibiotics13111042 39596736 PMC11591436

[98] Zheng H Xu Y Liehn EA Rusu M. Vitamin C as scavenger of reactive oxygen species during healing after myocardial infarction. Int J Mol Sci. (2024) 25(6):3114. 10.3390/ijms25063114 38542087 PMC10970003

[99] Ali HM Karam K Khan T Wahab S Ullah S Sadiq M. Reactive oxygen species induced oxidative damage to DNA, lipids, and proteins of antibiotic-resistant bacteria by plant-based silver nanoparticles. 3 Biotech. (2023) 13(12):414. 10.1007/s13205-023-03835-1 38009163 PMC10665289

[100] Alharthi S Ziora ZM Janjua T Popat A Moyle PM. Formulation and biological evaluation of mesoporous silica nanoparticles loaded with combinations of sortase A inhibitors and antimicrobial peptides. Pharmaceutics. (2022) 14(5):986. 10.3390/pharmaceutics14050986 35631572 PMC9144937

[101] Hatae AC Roque-Borda CA Pavan FR. Strategies for lipid-based nanocomposites with potential activity against Mycobacterium tuberculosis: microbial resistance challenge and drug delivery trends. OpenNano. (2023) 13:100171. 10.1016/j.onano.2023.100171

[102] Edlund A Yang Y Yooseph S He X Shi W McLean JS. Uncovering complex microbiome activities via metatranscriptomics during 24 h of oral biofilm assembly and maturation. Microbiome. (2018) 6(1):217. 10.1186/s40168-018-0591-4 30522530 PMC6284299

[103] Bhatt AH Patel HP Patel PR Rathod HG Hirawala JH Shaikh PS. Cubosomes in non-oral drug delivery: advancing precision therapeutics from bench to bedside. Int J Pharm. (2025) 684:126108. 10.1016/j.ijpharm.2025.126108 40865589

[104] Lakic B Beh C Sarkar S Yap SL Cardoso P Valery C. Cubosome lipid nanocarriers for delivery of ultra-short antimicrobial peptides. J Colloid Interface Sci. (2025) 677:1080–97. 10.1016/j.jcis.2024.07.232 39137610

[105] Canales CSC Cazorla JIM Cazorla RMM Sábio RM Santos HA Pavan FR. Combating gram-negative infections: the role of antimicrobial peptides and nanotechnology in overcoming antibiotic resistance. Materials Today Bio. (2025) 35:102381. 10.1016/j.mtbio.2025.102381 41142413 PMC12546895

[106] Saleem N Kumar N El-Omar E Willcox M Jiang XT. Nano-antimicrobial peptides (nano-AMPs) to combat resistant gram-negative bacteria. Drug Deliv Transl Res. (2026):1–35. 10.1007/s13346-026-02085-x 41772350 PMC13619782

[107] Maleki Dizaj S Salatin S Khezri K Lee J-Y Lotfipour F. Targeting multidrug resistance with antimicrobial peptide-decorated nanoparticles and polymers. Front Microbiol. (2022) 13:831655. 10.3389/fmicb.2022.831655 35432230 PMC9009044

[108] Palanikumar L Al-Hosani S Kalmouni M Nguyen VP Ali L Pasricha R. pH-responsive high stability polymeric nanoparticles for targeted delivery of anticancer therapeutics. Commun Biol. (2020) 3(1):95. 10.1038/s42003-020-0817-4 32127636 PMC7054360

[109] Rata DM Cadinoiu AN Atanase LI Popa M Mihai CT Vochita G. Peptide-functionalized chitosan-based microcapsules for dual active targeted treatment of lung infections. Int J Biol Macromol. (2024) 265:131027. 10.1016/j.ijbiomac.2024.131027 38518936

[110] Cadinoiu AN Rata DM Atanase LI Ichim DL Gherghel D Condriuc IP. Physicochemical characterization and *in vitro* evaluation of peptide-functionalized microspheres based on carboxymethyl chitosan and poly (vinyl alcohol) as promising pulmonary drug delivery system. Applied Materials Today. (2025) 44:102778. 10.1016/j.apmt.2025.102778

[111] Recktenwald M Kaur M Benmassaoud MM Copling A Khanna T Curry M. Antimicrobial peptide screening for designing custom bactericidal hydrogels. Pharmaceutics. (2024) 16(7):860. 10.3390/pharmaceutics16070860 39065557 PMC11279943

[112] Tang L Wang L Yang X Feng Y Li Y Feng W. Poly (N-isopropylacrylamide)-based smart hydrogels: design, properties and applications. Prog Mater Sci. (2021) 115:100702. 10.1016/j.pmatsci.2020.100702

[113] Ansari MJ Rajendran RR Mohanto S Agarwal U Panda K Dhotre K. Poly (N-isopropylacrylamide)-based hydrogels for biomedical applications: a review of the state-of-the-art. Gels. (2022) 8(7):454. 10.3390/gels8070454 35877539 PMC9323937

[114] Haider A Khan S Iqbal DN Shrahili M Haider S Mohammad K. Advances in chitosan-based drug delivery systems: a comprehensive review for therapeutic applications. Eur Polym J. (2024) 210:112983. 10.1016/j.eurpolymj.2024.112983

[115] Caracciolo G Palchetti S Digiacomo L Chiozzi RZ Capriotti AL Amenitsch H. Human biomolecular corona of liposomal doxorubicin: the overlooked factor in anticancer drug delivery. ACS Appl Mater Interfaces. (2018) 10(27):22951–62. 10.1021/acsami.8b04962 29905462

[116] Monopoli MP Walczyk D Campbell A Elia G Lynch I Baldelli Bombelli F. Physical-chemical aspects of protein corona: relevance to *in vitro* and *in vivo* biological impacts of nanoparticles. J Am Chem Soc. (2011) 133(8):2525–34. 10.1021/ja107583h 21288025

[117] Hu WW Lin ZW Ruaan RC Chen WY Jin SLC Chang Y. A novel application of indolicidin for gene delivery. Int J Pharm. (2013) 456(2):293–300. 10.1016/j.ijpharm.2013.08.035 23999223

[118] Hu WW Yeh CC Tsai CW. The conjugation of indolicidin to polyethylenimine for enhanced gene delivery with reduced cytotoxicity. J Mater Chem B. (2018) 6(36):5781–94. 10.1039/c8tb01408f 32254985

[119] Tsai CW Lin ZW Chang WF Chen YF Hu W-W. Development of an indolicidin-derived peptide by reducing membrane perturbation to decrease cytotoxicity and maintain gene delivery ability. Colloids and Surfaces B: Biointerfaces. (2018) 165:18–27. 10.1016/j.colsurfb.2018.02.007 29448216

[120] Hu WW Huang SC Jin SLC. A novel antimicrobial peptide-derived vehicle for oligodeoxynucleotide delivery to inhibit TNF-α expression. Int J Pharm. (2019) 558:63–71. 10.1016/j.ijpharm.2018.12.082 30639220

[121] Food, Drug Administration. Drug Products, Including Biological Products, that Contain Nanomaterials: Guidance for Industry. (Docket No FDA-2017-D-0759). (2022).

[122] European Medicines Agency. Guideline on the Development and Manufacture of Synthetic Peptides. (EMA/CHMP/CVMP/QWP/367182/2025). (2025).

[123] Doak SH Dusinska M. Nanogenotoxicology: present and the future. Mutagenesis. (2017) 32(1):1–4. 10.1093/mutage/gew066 28011747

[124] Dussert F Wegner KD Moriscot C Gallet B Jouneau PH Reiss P. Evaluation of the dermal toxicity of InZnP quantum dots before and after accelerated weathering: toward a safer-by-design strategy. Frontiers in Toxicology. (2021) 3:636976. 10.3389/ftox.2021.636976 35295141 PMC8915823

[125] Gupta A Landis RF Rotello VM. Nanoparticle-based antimicrobials: surface functionality is critical. F1000Res. (2016) 5(F1000-Faculty):364. 10.12688/f1000research.7595.1 PMC4798155 27006760

[126] Nafee N. Nanocarriers against bacterial biofilms: Current status and future perspectives. Nanotechnology in diagnosis, treatment and prophylaxis of infectious diseases, 167-189. (2015). 10.1016/B978-0-12-801317-5.00011-6

[127] Griffin BT Guo J Presas E Donovan MD Alonso MJ. Pharmacokinetic, pharmacodynamic and biodistribution following oral administration of nanocarriers containing peptide and protein drugs. Adv Drug Delivery Rev. (2016) 106:367–80. 10.1016/j.addr.2016.06.006 27320644

[128] Yang YX Metz DC. Safety of proton pump inhibitor exposure. Gastroenterology. (2010) 139(4):1115–27. 10.1053/j.gastro.2010.08.023 20727892

[129] Thakkar N Salerno S Hornik CP Gonzalez D. Clinical pharmacology studies in critically ill children: thakkar, salerno, hornik and gonzalez. Pharm Res. (2017) 34(1):7–24. 10.1007/s11095-016-2033-y 27585904 PMC5177463

[130] Baracaldo-Santamaría D Cala-Garcia JD Medina-Rincón GJ Rojas-Rodriguez LC Calderon-Ospina CA. Therapeutic drug monitoring of antifungal agents in critically ill patients: is there a need for dose optimisation? Antibiotics. (2022) 11(5):645. 10.3390/antibiotics11050645 35625289 PMC9137962

[131] Bonilla FA Barlan I Chapel H Costa-Carvalho BT Cunningham-Rundles C de la Morena MT. International consensus document (ICON): common variable immunodeficiency disorders. The Journal of Allergy and Clinical Immunology in Practice. (2015) 4(1):38. 10.1016/j.jaip.2015.07.025 26563668 PMC4869529

[132] He W Fu D Gai Y Liu X Yang C Ye Z. An infection-microenvironment-targeted and responsive peptide-drug nanosystem for sepsis emergency by suppressing infection and inFlammation. Asian J Pharmaceut Sci. (2023) 18(6):100869. 10.1016/j.ajps.2023.100869 PMC10755722 38161786

[133] León-Buitimea A Garza-Cárdenas CR Garza-Cervantes JA Lerma-Escalera JA Morones-Ramírez JR. The demand for new antibiotics: antimicrobial peptides, nanoparticles, and combinatorial therapies as future strategies in antibacterial agent design. Front Microbiol. (2020) 11:1669. 10.3389/fmicb.2020.01669 32793156 PMC7393301

[134] Hetta HF Ramadan YN Al-Harbi AIA Ahmed E Battah B Abd Ellah NH. Nanotechnology as a promising approach to combat multidrug resistant bacteria: a comprehensive review and future perspectives. Biomedicines. (2023) 11:413. 10.3390/biomedicines11020413 36830949 PMC9953167

[135] Häffner SM Nyström L Nordström R Xu ZP Davoudi M Schmidtchen A. Membrane interactions and antimicrobial effects of layered double hydroxide nanoparticles. Phys Chem Chem Phys. (2017) 19(35):23832–42. 10.1039/c7cp02701j 28682360

[136] Allahverdiyev AM Kon KV Abamor ES Bagirova M Rafailovich M. Coping with antibiotic resistance: combining nanoparticles with antibiotics and other antimicrobial agents. Expert Rev Anti-Infect Ther. (2011) 9(11):1035–52. 10.1586/eri.11.121 22029522

[137] Chou TC. Theoretical basis, experimental design, and computerized simulation of synergism and antagonism in drug combination studies. Pharmacol Rev. (2006) 58(3):621–81. 10.1124/pr.58.3.10 16968952

[138] Caleffi-Ferracioli KR Maltempe FG Siqueira VLD Cardoso RF. Fast detection of drug interaction in Mycobacterium tuberculosis by a checkerboard resazurin method. Tuberculosis. (2013) 93(6):660–3. 10.1016/j.tube.2013.09.001 24083948

[139] Gurvic D Zachariae U. Multidrug efflux in gram-negative bacteria: structural modifications in active compounds leading to efflux pump avoidance. NPJ Antimicrobials and Resistance. (2024) 2(1):6. 10.1038/s44259-024-00023-w 39843816 PMC11721645

[140] Blair JM Piddock LJ. How to measure export via bacterial multidrug resistance efflux pumps. MBio. (2016) 7(4):10–1128. 10.1128/mBio.00840-16 PMC4958252 27381291

[141] Lomovskaya O Bostian KA. Practical applications and feasibility of efflux pump inhibitors in the clinic—a vision for applied use. Biochem Pharmacol. (2006) 71(7):910–8. 10.1016/j.bcp.2005.12.008 16427026

[142] Anselmo AC Mitragotri S. Nanoparticles in the clinic: an update post COVID-19 vaccines. Bioeng Transl Med. (2021) 6(3):e10246. 10.1002/btm2.10246 34514159 PMC8420572

[143] Mitchell MJ Billingsley MM Haley RM Wechsler ME Peppas NA Langer R. Engineering precision nanoparticles for drug delivery. Nat Rev Drug Discov. (2021) 20(2):101–24. 10.1038/s41573-020-0090-8 33277608 PMC7717100

[144] Shreffler JW Pullan JE Dailey KM Mallik S Brooks AE. Overcoming hurdles in nanoparticle clinical translation: the influence of experimental design and surface modification. Int J Mol Sci. (2019) 20(23):6056. 10.3390/ijms20236056 31801303 PMC6928924

[145] Calvert ND Yu L Sehl OC Gevaert JJ Knier NN Rivera-Rodriguez A. The careful selection of zwitterionic nanoparticle coating results in rapid and efficient cell labeling for imaging-based cell tracking. Aggregate. (2024) 5(6):e609. 10.1002/agt2.609 PMC12478535 41030921

[146] Bertrand N Grenier P Mahmoudi M Lima EM Appel EA Dormont F. Mechanistic understanding of *in vivo* protein corona formation on polymeric nanoparticles and impact on pharmacokinetics. Nat Commun. (2017) 8(1):777. 10.1038/s41467-017-00600-w 28974673 PMC5626760

[147] Panagi Z Beletsi A Evangelatos G Livaniou E Ithakissios DS Avgoustakis K. Effect of dose on the biodistribution and pharmacokinetics of PLGA and PLGA–mPEG nanoparticles. Int J Pharm. (2001) 221(1-2):143–52. 10.1016/S0378-5173(01)00676-7 11397575

[148] Zhou C Liu Y Li Y Shi L, Recent advances and prospects in nanomaterials for bacterial sepsis management. J Mater Chem B. (2023) 11(45):10778–92. 10.1039/D3TB02220J 37901894

[149] Ozbek O Genc DE Ulgen KO. Advances in physiologically based pharmacokinetic (PBPK) modeling of nanomaterials. ACS Pharmacol Transla Sci. (2024) 7(8):2251–79. 10.1021/acsptsci.4c00250 PMC11320736 39144562

[150] Danaei M Dehghankhold M Ataei S Hasanzadeh Davarani F Javanmard R Dokhani A. Impact of particle size and polydispersity index on the clinical applications of lipidic nanocarrier systems. Pharmaceutics. (2018) 10(2):57. 10.3390/pharmaceutics10020057 29783687 PMC6027495

[151] Lee SH Jun BH. Silver nanoparticles: synthesis and application for nanomedicine. Int J Mol Sci. (2019) 20:865. 10.3390/ijms20040865 30781560 PMC6412188

[152] Ghosh R De M. Liposome-based antibacterial delivery: an emergent approach to combat bacterial infections. ACS Omega. (2023) 8(39):35442–51. 10.1021/acsomega.3c04893 37810644 PMC10551917

[153] Siramshetty VB Grishagin I Nguyễn ĐT Peryea T Skovpen Y Stroganov O. NCATS Inxight drugs: a comprehensive and curated portal for translational research. Nucleic Acids Res. (2022) 50(D1):D1307–16. 10.1093/nar/gkab918 34648031 PMC8728186

[154] Chaudhary S Ali Z Tehseen M Haney EF Pantoja-Angles A Alshehri S. Efficient in planta production of amidated antimicrobial peptides that are active against drug-resistant ESKAPE pathogens. Nat Commun. (2023) 14(1):1464. 10.1038/s41467-023-37003-z 36928189 PMC10020429

[155] Fiegler-Rudol J Lipka B Kapłon K Mos M Skaba D Kawczyk-Krupka A. Evaluating the efficacy of rose Bengal as a photosensitizer in antimicrobial photodynamic therapy against *Candida albicans*: a systematic review. Int J Mol Sci. (2025) 26(11):5034. 10.3390/ijms26115034 40507862 PMC12154552

[156] Mahmoudi H Bahador A Pourhajibagher M Alikhani MY. Antimicrobial photodynamic therapy: an effective alternative approach to control bacterial infections. J Lasers Med Sci. (2018) 9(3):154. 10.15171/jlms.2018.29 30809325 PMC6378356

[157] Dong R Liu R Liu Z Liu Y Zhao G Li H. Exploring the repository of *de novo*-designed bifunctional antimicrobial peptides through deep learning. eLife. (2025) 13:RP97330. 10.7554/eLife.97330.2 40079572 PMC11906162

[158] Wang GS Li X Wang Z. APD3: the antimicrobial peptide database as a tool for research and education. Nucleic Acids Res. (2016) 44:D1087–93. 10.1093/nar/gkv1278 26602694 PMC4702905

[159] Pirtskhalava M Amstrong AA Grigolava M Chubinidze M Alimbarashvili E Vishnepolsky B. DBAASP V3: database of antimicrobial/cytotoxic activity and structure of peptides as a resource for development of new therapeutics. Nucleic Acids Res. (2021) 49(D1):D288–97. 10.1093/nar/gkaa991 33151284 PMC7778994

[160] Veltri D Kamath U Shehu A. Deep learning improves antimicrobial peptide recognition. Bioinformatics. (2018) 34(16):2740–7. 10.1093/bioinformatics/bty179 29590297 PMC6084614

[161] Waghu FH Gopi L Barai RS Ramteke P Nizami B Idicula-Thomas S. CAMP: collection of sequences and structures of antimicrobial peptides. Nucleic Acids Res. (2014) 42(D1):D1154–8. 10.1093/nar/gkt1157 24265220 PMC3964954

[162] Al Musaimi, O. FDA-Approved Antibacterials and echinocandins. Antibiotics. (2025) 14(2):166. 10.3390/antibiotics14020166 40001410 PMC11851826

[163] Arsene MM Jorelle AB Sarra S Viktorovna PI Davares AK Ingrid NK. Short review on the potential alternatives to antibiotics in the era of antibiotic resistance. J Appl Pharm Sci. (2022) 12(7):29–40. 10.7324/JAPS.2021.120102

[164] Bagheri M Beyermann M Dathe M. Mode of action of cationic antimicrobial peptides defines the tethering position and the efficacy of biocidal surfaces. Bioconjugate Chem. (2012) 23(1):66–74. 10.1021/bc200367f 22148269

[165] Bayón-Cordero L Alkorta I Arana L. Application of solid lipid nanoparticles to improve the efficiency of anticancer drugs. Nanomaterials. (2019) 9(3):474. 10.3390/nano9030474 30909401 PMC6474076

[166] Deng H Zhang S Fu Y Dong N Bi C Shan A. Advances in the delivery and application of antimicrobial peptide-based nanomaterials. Chem Eng J. (2024) 496:154232. 10.1016/j.cej.2024.154232

[167] Santos N D Allen C Doppen AM Anantha M Cox KA Gallagher RC. Influence of poly (ethylene glycol) grafting density and polymer length on liposomes: relating plasma circulation lifetimes to protein binding. Biochim Biophys Acta. (2007) 1768(6):1367–77. 10.1016/j.bbamem.2006.12.013 17400180

[168] Drayton M Kizhakkedathu JN Straus SK. Towards robust delivery of antimicrobial peptides to combat bacterial resistance. Molecules. (2020) 25(13):3048. 10.3390/molecules25133048 32635310 PMC7412191

[169] Eleraky NE Allam A Hassan SB Omar MM. Nanomedicine fight against antibacterial resistance: an overview of the recent pharmaceutical innovations. Pharmaceutics. (2020) 12(2):142. 10.3390/pharmaceutics12020142 32046289 PMC7076477

[170] Eliopoulos GM Moellering RC Jr. Antibiotic synergism and antimicrobial combinations in clinical infections george. Rev Infect Dis. (1982) 4(2):282–93. 10.1093/clinids/4.2.282 7051231

[171] EMA. European Medicines Agency (2006, revisado 2025) Guideline on ERA of medicinal products, EMA/CHMP/SWP/4447/00).

[172] Fadaka AO Sibuyi NRS Madiehe AM Meyer M. Nanotechnology-based delivery systems for antimicrobial peptides. Pharmaceutics. (2021) 13(11):1795. 10.3390/pharmaceutics13111795 34834210 PMC8620809

[173] Falanga A Del Genio V Galdiero S. Peptides and dendrimers: how to combat viral and bacterial infections. Pharmaceutics. (2021) 13(1):101. 10.3390/pharmaceutics13010101 33466852 PMC7830367

[174] Farzin A Etesami SA Quint J Memic A Tamayol A. Magnetic nanoparticles in cancer therapy and diagnosis. Adv Healthcare Mater. (2020) 9(9):1901058. 10.1002/adhm.201901058 PMC7482193 32196144

[175] Gide M Nimmagadda A Su M Wang M Teng P Li C. Nano-Sized lipidated dendrimers as potent and broad-Spectrum antibacterial agents. Macromol Rapid Commun. (2018) 39:e1800622. 10.1002/marc.201800622 30408252

[176] Harini K Girigoswami K Thirumalai A Girigoswami A. Polymer-based antimicrobial peptide mimetics for treating multi-drug resistant infections: therapy and toxicity evaluation. Int J Pept Res Ther. (2024) 30. 10.1007/s10989-024-10648-039465062

[177] Huan Y Kong Q Mou H Yi H. Antimicrobial peptides: classification, design, application and research progress in multiple fields. Front Microbiol. (2020) 11:582779. 10.3389/fmicb.2020.582779 33178164 PMC7596191

[178] Hulugalla K Shofolawe-Bakare O Toragall VB Mohammad SA Mayatt R Hand K. Glycopolymeric nanoparticles enrich less immunogenic protein coronas, reduce mononuclear phagocyte clearance, and improve tumor delivery compared to PEGylated nanoparticles. ACS Nano. (2024) 18(44):30540–60. 10.1021/acsnano.4c08922 39436672 PMC12045476

[179] Johnson TS Bourdine AA Deber CM. Hydrophobic moment drives penetration of bacterial membranes by transmembrane peptides. J Biol Chem. (2023) 299(11):105266. 10.1016/j.jbc.2023.105266 37734555 PMC10585379

[180] Kommineni N Mahira S Domb AJ Khan W. Cabazitaxel-loaded nanocarriers for cancer therapy with reduced side effects. Pharmaceutics. (2019) 11(3):141. 10.3390/pharmaceutics11030141 30934535 PMC6470818

[181] Kumari A Yadav SK Yadav SC. Biodegradable polymeric nanoparticles based drug delivery systems. Colloids Surf B Biointerfaces. (2010) 75(1):1–18. 10.1016/j.colsurfb.2009.09.001 19782542

[182] Laverty G McCloskey AP Gilmore BF Jones DS Zhou J Xu B. Ultrashort cationic naphthalene-derived self-assembled peptides as antimicrobial nanomaterials. Biomacromolecules. (2014) 15(9):3429–39. 10.1021/bm500981y 25068387

[183] Lazaro E Kadie C Stamegna P Zhang SC Gourdain P Lai NY. Variable HIV peptide stability in human cytosol is critical to epitope presentation and immune escape. J Clin Invest. (2011) 121(6):2480–92. 10.1172/JCI44932 21555856 PMC3104749

[184] Lopez-Silva TL Leach DG Li IC Wang X Hartgerink JD. Self-assembling multidomain peptides: design and characterization of neutral peptide-based materials with pH and ionic strength independent self-assembly. ACS Biomater Sci Eng. (2019) 5:977–85. 10.1021/acsbiomaterials.8b01348 31404449 PMC6688848

[185] Makadia HK Siegel SJ. Poly lactic-co-glycolic acid (PLGA) as biodegradable controlled drug delivery carrier. Polymers (Basel). (2011) 3(3):1377–97. 10.3390/polym3031377 22577513 PMC3347861

[186] Meade E Slattery MA Garvey M. Bacteriocins, potent antimicrobial peptides and the fight against multi drug resistant species: resistance is futile? Antibiotics. (2020) 9(1):32. 10.3390/antibiotics9010032 31963311 PMC7168330

[187] Mi P. Stimuli-responsive nanocarriers for drug delivery, tumor imaging, therapy and theranostics. Theranostics. (2020) 10(10):4557. 10.7150/thno.38069 32292515 PMC7150471

[188] OECD. Test No. 476: In Vitro Mammalian Cell Gene Mutation Tests Using the Hprt and Xprt Genes. OECD Guidelines for the Testing of Chemicals, Section 4. Paris: OECD Publishing (2016). 10.1787/9789264264809-en

[189] OECD. Test No. 487: In Vitro Mammalian Cell Micronucleus Test. OECD Guidelines for the Testing of Chemicals, Section 4. Paris: OECD Publishing (2023). 10.1787/9789264264861-en

[190] Roque-Borda CA Bento da Silva P Rodrigues MC Di Filippo LD Duarte JL Chorilli M. Pharmaceutical nanotechnology: antimicrobial peptides as potential new drugs against WHO list of critical, high, and medium priority bacteria. Eur J Med Chem. (2022) 241:114640. 10.1016/j.ejmech.2022.114640 35970075

[191] Roy S Hasan I Guo B. Recent advances in nanoparticle-mediated antibacterial applications. Coord Chem Rev. (2023) 482:215075. 10.1016/j.ccr.2023.215075

[192] Tsai CW Hsu NY Wang CH Lu CY Chang Y Tsai HHG. Coupling molecular dynamics simulations with experiments for the rational design of indolicidin-analogous antimicrobial peptides. J Mol Biol. (2009) 392(3):837–54. 10.1016/j.jmb.2009.06.071 19576903

[193] Wang T Ji L Zhang Y Niu Z Jiang X Wang X. Antimicrobial peptide nanoassemblies: design, response mechanisms, and biomedical applications. Molecules. (2026) 31(3):518. 10.3390/molecules31030518 41683494 PMC12899442

[194] Werengowska-Ciećwierz K Wiśniewski M Terzyk AP Furmaniak S. The chemistry of bioconjugation in nanoparticles-based drug delivery system. Advances in Condensed Matter Physics. (2015) 2015:198175. 10.1155/2015/198175

[195] Zhou X Tao H Shi KH. RETRACTED ARTICLE: development of a nanoliposomal formulation of erlotinib for lung cancer and *in vitro*/*in vivo* antitumoral evaluation. Drug Des Devel Ther. (2017) 12:1–8. 10.2147/DDDT.S146925 29296076 PMC5739116

[196] Murray CJL Ikuta KS Sharara F Swetschinski L Robles Aguilar G Gray A. Global burden of bacterial antimicrobial resistance in 2019: a systematic analysis. Lancet. (2022) 399(10325):629–55. 10.1016/S0140-6736(21)02724-0 35065702 PMC8841637

[197] Cavaco M Andreu D Castanho MARB. The challenge of peptide proteolytic stability studies: scarce data, difficult readability, and the need for harmonization. Angew Chem, Int Ed. (2021) 60(4):1686–8. 10.1002/anie.202006372 33200441

[198] Bera S Ghosh A Sharma S Debnath T Giri B Bhunia A. Probing the role of proline in the antimicrobial activity and lipopolysaccharide binding of indolicidin. J Colloid Interface Sci. (2015) 452:148–59. 10.1016/j.jcis.2015.04.031 25935286

[199] Bowdish DME Davidson DJ Lau YE Lee K Scott MG Hancock REW. Impact of LL-37 on anti-infective immunity. J Leukocyte Biol. (2005) 77(4):451–9. 10.1189/jlb.0704380 15569695

[200] Ghaffar KA Hussein WM Khalil ZG Capon RJ Skwarczynski M Toth I. Levofloxacin and indolicidin for combination antimicrobial therapy. Curr Drug Delivery. (2015) 12(1):108–14. 10.2174/1567201811666140910094050 25213074

[201] Lei J Sun L Huang S Zhu C Li P He J. The antimicrobial peptides and their potential clinical applications. Am J Transl Res. (2019) 11(7):3919–31. https://pubmed.ncbi.nlm.nih.gov/31396309/31396309 PMC6684887

[202] Hu X Gong H Liao M Lu JR. Short peptide supramolecular hydrogels for antimicrobial applications. In: Yan X, editor. Peptide Self-assembly and Engineering. Weinheim: Wiley-VCH (2024). p. 465–94. 10.1002/9783527841264.ch19

[203] Mazur P Skiba-Kurek I Mrowiec P Karczewska E Drożdż R. Synergistic ROS-associated antimicrobial activity of silver nanoparticles and gentamicin against *Staphylococcus epidermidis*. Int J Nanomed. (2020) 15:3551–62. 10.2147/IJN.S246484 PMC7246328 32547013

[204] Altevogt BM Taylor P Akwar HT Graham DW Ogilvie LA Duffy E. A one health framework for global and local stewardship across the antimicrobial lifecycle. Communications Medicine. (2025) 5(1):414. 10.1038/s43856-025-01090-4 41057683 PMC12504729

[205] Allen TM Cullis PR. Drug delivery systems: entering the mainstream. Science. (2004) 303(5665):1818–22. 10.1126/science.1095833 15031496

[206] Yuan D He H Wu Y Fan J Cao Y. Physiologically based pharmacokinetic modeling of nanoparticles. J Pharm Sci. (2019) 108(1):58–72. 10.1016/j.xphs.2018.10.037 30385282 PMC6311421

[207] Bera A Singh S Nagaraj R Vaidya T. Induction of autophagic cell death in *Leishmania donovani* by antimicrobial peptides. Mol Biochem Parasitol. (2003) 127(1):23–35. 10.1016/S0166-6851(02)00300-6 12615333

[208] Aley SB Zimmerman M Hetsko M Selsted ME Gillin FD. Killing of *Giardia lamblia* by cryptdins and cationic neutrophil peptides. Infect Immun. (1994) 62(12):5397–403. 10.1128/iai.62.12.5397-5403.1994 7960119 PMC303280

